# Metagenomic analysis reveals the microbiome and resistome in migratory birds

**DOI:** 10.1186/s40168-019-0781-8

**Published:** 2020-03-02

**Authors:** Jian Cao, Yongfei Hu, Fei Liu, Yanan Wang, Yuhai Bi, Na Lv, Jing Li, Baoli Zhu, George F. Gao

**Affiliations:** 1 CAS Key Laboratory of Pathogenic Microbiology and Immunology, Institute of Microbiology, Chinese Academy of Sciences, Beijing, China; 2 Savaid Medical School, University of Chinese Academy of Sciences, Beijing, China; 3 State Key Laboratory of Animal Nutrition, College of Animal Science and Technology, China Agricultural University, Beijing, China; 4 National Institute for Communicable Disease Control and Prevention, Chinese Center for Disease Control and Prevention, Beijing, China

**Keywords:** Microbiome, Resistome, Antibiotic resistance, Antibiotic resistance genes (ARGs), β-Lactamase, Migratory birds

## Abstract

**Background:**

Antibiotic-resistant pathogens pose high risks to human and animal health worldwide. In recent years, the role of gut microbiota as a reservoir of antibiotic resistance genes (ARGs) in humans and animals has been increasingly investigated. However, the structure and function of the gut bacterial community, as well as the ARGs they carry in migratory birds remain unknown.

**Results:**

Here, we collected samples from migratory bird species and their associated environments and characterized their gut microbiomes and resistomes using shotgun metagenomic sequencing. We found that migratory birds vary greatly in gut bacterial composition but are similar in their microbiome metabolism and function. Birds from the same environment tend to harbor similar bacterial communities. In total, 1030 different ARGs (202 resistance types) conferring resistance to tetracycline, aminoglycoside, β-lactam, sulphonamide, chloramphenicol, macrolide-lincosamide-streptogramin (MLS), and quinolone are identified. Procrustes analysis indicated that microbial community structure is not correlated with the resistome in migratory birds. Moreover, metagenomic assembly-based host tracking revealed that most of the ARG-carrying contigs originate from *Proteobacteria*. Co-occurrence patterns revealed by network analysis showed that *emrD*, *emrY*, *ANT(6)-Ia*, and *tetO*, the hubs of ARG type network, are indicators of other co-occurring ARG types. Compared with the microbiomes and resistomes in the environment, migratory birds harbor a lower phylogenetic diversity but have more antibiotic resistance proteins. Interestingly, we found that the *mcr-1* resistance gene is widespread among different birds, accounting for 50% of the total samples. Meanwhile, a large number of novel β-lactamase genes are also reconstructed from bird metagenomic assemblies based on fARGene software.

**Conclusions:**

Our study provides a comprehensive overview of the diversity and abundance of ARGs in migratory birds and highlights the possible role of migratory birds as ARG disseminators into the environment.

Video abstract.

## Introduction

The coevolution of vertebrates and their associated microbes can improve the adaptability of a host to new dietary niches [1, 2]. An increasing amount of data, from humans and mice to insects and fish, has provided the insight that microbial communities colonizing the gut play a critical role in affecting host physiology and health [3]. These communities are capable of extracting nutrients from ingested food and producing metabolic bioactive molecules for the host. Comparing with the host genome, a range of factors can influence the gut microbial structure. An analysis from humans and 59 other mammals’ microbiota revealed that host genetics and diet are believed to be the two major drivers of a host’s gut microbial structure [1]. In addition, functional configurations of microbiomes are closely linked to gut microbial assemblages across different mammalian lineages [2].

Approximately 700,000 people die annually due to resistant infections, and this number will increase to 10 million annual deaths by the year 2050 [4, 5]. Environments, including soil, water, and animal waste, harbor abundant and diverse antibiotic resistance genes (ARGs) and contribute to the spread of natural resistomes into bacteria through mechanisms such as horizontal gene transfer (HGT) [6]. The gut microbiota is a collection of microorganisms living in the gastrointestinal tract of the host. This community consists of eukaryotes, archaea, bacteria, and viruses, which are essential for maintaining host health. However, a growing body of direct and indirect studies reveals that the gut microbiome can act as reservoir for antibiotic-resistant bacteria (ARB) and their associated ARGs [7–9]. Metagenome-wide analyses of both human and pig gut microbiota show that representative resistance gene types from humans in different countries are distinctive and may be attributed to country-specific antibiotic use practices [7]. The gut microbial members of pigs also carry ARBs with mobile genetic elements (MGEs) belonging to diverse genotypes [8]. Antimicrobial resistance (AMR) is not just a regional or national problem; a number of factors can lead to their rapid dissemination across the world. For example, the *Enterobacteriaceae* with *Klebsiella pneumoniae* carbapenemase (*KPC*), New Delhi Metallo-β-lactamase (*NDM*), and *mcr-1* have been transmitted from their locations/country of origin to Asia, Europe, and North America through international human travel and the food-supply chain [10]. In addition, wild animals might play another key role as a biological mechanism for the development and intercontinental dissemination of both ARBs and ARGs [11].

The class Aves contains over ~ 10,500 living species that occupy a wide range of niches throughout the world [12]. Birds, especially waterfowl, can migrate long geographical distances between their breeding and wintering areas, and they can develop complex physiological traits and unique diets and lifestyles to live in diverse natural environments like remote mountain lakes [11]. Comparing with other vertebrates, the whole genomes of 48 avian species representing all orders of Neoaves are of constrained size [13]. Like vertebrates, trillions of microbes live in the guts of birds and aid in nutrition, immunity, and gut development of their hosts [14]. Furthermore, several studies demonstrate that migratory birds both carry and acquire ARBs from contaminated environments near areas with high livestock or human densities and transmit the bacteria along migration flyways [15]. However, little attention has been paid to the gut microbial composition and its associated ARGs in migrating birds, in contrast to the extensive studies of human and mouse microbiomes and resistomes. Consequently, a comprehensive study on the structure and function of the bacterial composition and its associated ARGs in migratory wild birds is urgently needed.

Although scientists have conducted inventories of the structure of intestinal microbiota from several avian species through 16S rRNA data (e.g., hoatzin, kakapo, penguins, vultures, seabirds, and hooded cranes [16]), we know little about the functional repertoires of bird gut microbiome genes. ARBs from wild birds have been previously studied by different methods, including bacterial culturing and molecular tools of PCR or qPCR [17], which have identified *Escherichia coli* as the most prevalent resistant bacteria in ducks and geese, cormorants, gulls, doves, and passerines [18]. However, only limited microbes are cultivable and express their ARGs in vitro. PCR and qPCR also depend on available primers. Thus, these methods struggle to represent the overall information of ARGs in wild birds. Rapid, high-throughput metagenomics have recently been used to annotate functional pathways and identify ARGs in the microbiota of humans and soil samples. Here, we collected fresh samples from 10 bird species and their associated environments and used whole-metagenome shotgun sequencing to characterize their microbiomes and resistomes. We applied the metagenomic approach to achieve the following goals: (1) depict the gut microbial communities in migratory bird population, (2) uncover the metabolism and function of the gut microbiome among migratory birds, (3) detect the antibiotic resistome in migratory bird feces over a wide range of regions, (4) investigate the correlation between bacteria and resistomes, and (5) explore the differences between the microbiomes and resistomes of birds and their environment. This large survey of the microbial community and ARGs in migratory bird populations reflects the comprehensive resistome profiles and reveals the potential risks to human health caused by ARGs in migratory birds.

## Results

### Distinct gut bacterial communities in different migratory bird population

**Fig. 1:**
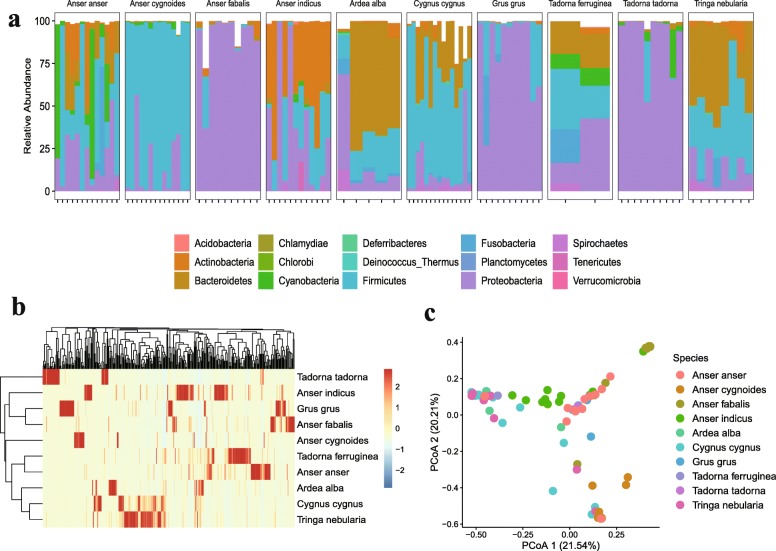
Comparison of gut bacterial composition among bird species. **a** Bacterial abundance. **b** Bacterial communities cluster analysis by heatmap based on Bray–Curtis distance. **c** Principal coordinate analysis (PCoA) based on Bray–Curtis distance

Bacterial units predominated in all bird species and contributed more phylogenetic diversity than archaea, eukaryotes, and viruses (Additional file 11: Table S3). At the phylum level, the sequenced reads from all samples were assigned to 15 different phyla and bird species differed markedly in their patterns of bacterial composition. For example, in *Grus grus*, *Anser fabalis*, and *Tadorna tadorna*, the most abundant phylum was *Proteobacteria* (82.4%, 89.2%, and 90.6%, respectively), while in *Cygnus cygnus* and *Anser cygnoides*, the most enriched phylum was *Firmicutes* (58.0% and 85.7%, respectively). *Alba alba* and *Tringa nebularia* had a higher proportion of *Bacteroidetes* (52.0% and 40.4%, respectively), and *Anser indicus* carried more abundant phylum *Actinobacteria* (40.5%). In general, *Proteobacteria*, *Firmicutes*, *Bacteroidetes*, and *Actinobacteria* were the four dominant phyla in all species, accounting for 75–98% of the total bacterial community (Fig. 1a). *Proteobacteria* was dominated by *Gammaproteobacteria*, *Betaproteobacteria*, and *Alphaproteobacteria* (Additional file 2: Figure S2a); *Firmicutes* was largely represented by *Clostridia* and *Bacilli* (Additional file 2: Figure S2b). *Bacteroida* and *Cytophagia* were the most abundant classes in the phylum *Bacteroidetes* (Additional file 2: Figure S2c); *Actinobacteria* consisted only of the class *Actinobacteria*.

At the genus level, 295 genera were identified across all samples, and the representative genera in each species were diverse. *Tadorna tadorna* had the uniquely enriched genus *Vibrio* (74.8%), whereas *Anser cygnoides* harbored the most abundant genus *Peptostreptococcaceae noname* (84.7%). A large proportion of the genus *Pseudomonas* was found in *Grus grus* and *Anser fabalis* species (55.8% and 64.8%, respectively), while the dominant genus *Bacteroides* was detected in *Alba alba* and *Tringa nebularia* species (51.2% and 37.2%, respectively). We identified 76 species-specific genera, in total, with a relatively low abundance, e.g., all *Anser* species (26), both *Tadorna* species (23), *Grus* species (13), *Cygnus* species (7), and *Tringa* species (3). Although only several genera were shared by all species, including *Pseudomonas*, *Peptostreptococcaceae_noname*, and *Escherichia*, the top 50 most abundant genera represented a large proportion of the microbial community in each bird population, ranging from 85 to 97%.

To distinguish between the variations of host gut microbiomes, the diversity indices (α-diversity and β-diversity) of microbial communities among different species were also evaluated (Additional file 3: Figure S3). The α-diversity indices, including observed genera and the Shannon index, demonstrated that the number of genera in *Tringa nebularia* was higher than in other species, whereas the Shannon index of the gut microbiota was significantly more diverse in *Anser indicus* and *Tadorna ferruginea* species. Furthermore, the results of the heatmap (Fig. 1b) and Principle Coordination Analysis (PCoA) based on the Bray–Curtis distance (Fig. 1c) revealed the dissimilarity of bacterial communities among species (adonis, *R*^2^ = 0.52, *P* < 0.001). Interestingly, samples from species living in the same geographic environments, (i.e., *Anser anser* and *Tadorna ferruginea* living at Qinghai Lake), tended to have similar gut microbial structures, which were not related to the genetic backgrounds of the hosts.

Approximately 15 opportunistic pathogens were detected in 50% of the species via comparison against a previously published pathogen list [19]. *E. coli* and *Fusobacterium mortiferum* were the most prevalent, and *Grus grus* (35) and *Anser fabalis* (30) species had the most diverse array of pathogens. Some clinically important pathogens were also observed in birds, such as *Clostridium perfringens*, *Klebsiella pneumoniae*, and *Clostridium difficile*.

### Similar metabolism and function of the gut microbiome among migratory birds

We then annotated the metabolic and functional pathways of the birds’ gut microbiome non-redundant (NR) gene catalog based on the eggNOG and KEGG databases. From eggNOG annotation results, we found that most of the gene functions remained unclear (28.6%), while replication, recombination, and repair (7.3%); amino acid transport and metabolism (6.7%); and carbohydrate transport and metabolism (6.3%) were relatively more abundant among the known functions (Additional file 4: Figure S4a). Meanwhile, KEGG annotation revealed that ~ 60% of the NR genes were classified into 12,425 KEGG orthology (KO) functions in total. The annotated functions showed similar trends among species, ranging from a maximum of only ~ 9185 functions per community in *Cygnus cygnus* to almost 3901 in *Anser fabalis*; 1182 related annotated functions were distributed in all individuals as core functions in the gut microbiome of birds. Those KO functions were mapped into 41 level II orthology groups (Additional file 4: Figure S4b). A large number of functions belonged to metabolism groups (61%), including carbohydrate metabolism (12.8%), energy metabolism (8.2%), amino acid metabolism (4.9%), nucleotide metabolism (4.1%), lipid metabolism (3.9%), xenobiotics biodegradation and metabolism (2.6%), and glycan biosynthesis and metabolism (2.1%).

**Fig. 2:**
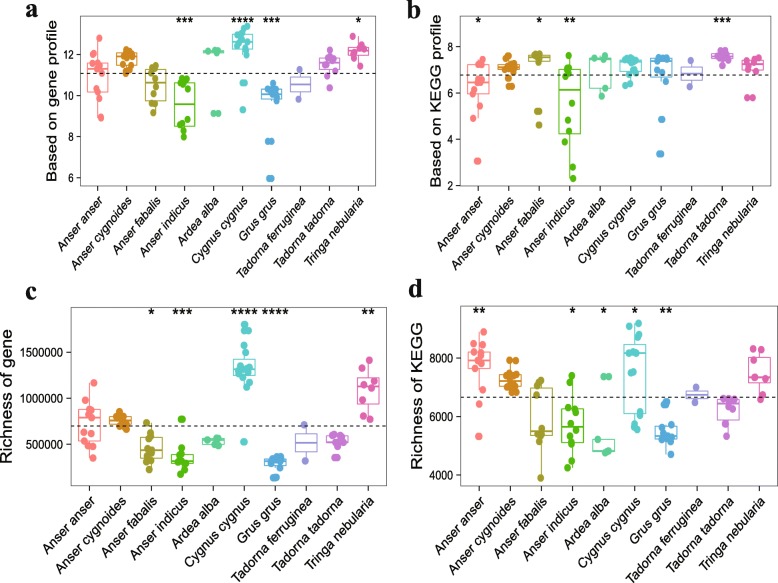
**a**–**d** Comparison of the gut microbiome gene catalogs among bird species. **a**–**b** α-diversity (Shannon index) and **c**–**d** richness. Boxes denote the interquartile (IQR) between the first and third quartiles (25th and 75th percentiles, respectively) and the line inside denotes the median. Whiskers denote the lowest and highest values within 1.5 times and the IQR from the first and third quartiles, respectively. The asterisks on the top indicate *P < 0.05, **P < 0.01, and ***P < 0.001, (Mann–Whitney *U* test)

We further compared α-diversity (the diversity within samples) and richness (the abundance within samples) in the individuals at the gene and KO levels (Fig. 2). *Cygnus cygnus* and *Tringa nebularia* species harbored higher gene α-diversity than the other species, while *Tadorna tadorna* was the most enriched in KEGG α-diversity among bird species. Regarding gene richness, *Tringa nebularia* and *Cygnus cygnus* exhibited higher abundance than the other bird species, whereas KO richness in *Anser anser* and *Cygnus cygnus* was higher than that observed in other species.

Compared to the wide variations in microbial communities among individuals, the relative abundance of their functional classification remained more stable and evenly diverse. Several metabolic pathways displayed broad prevalence and relatively abundant carriage patterns among species. Most of these “core” pathways were related to carbohydrate, energy, and amino acid metabolism. Pathways with low abundance and high variability were also present in populations, including sensory system, signaling molecules and interaction, cardiovascular diseases, and immune diseases. Interestingly, the functional pathways of lipid metabolism (7.1%) and transport and catabolism (4.2%) appeared to be more abundant in *Anser indicus* than other species, indicating a special interactions between the host and it gut microbes.

### Abundant ARGs in the gut bacteria of migratory birds

**Fig. 3:**
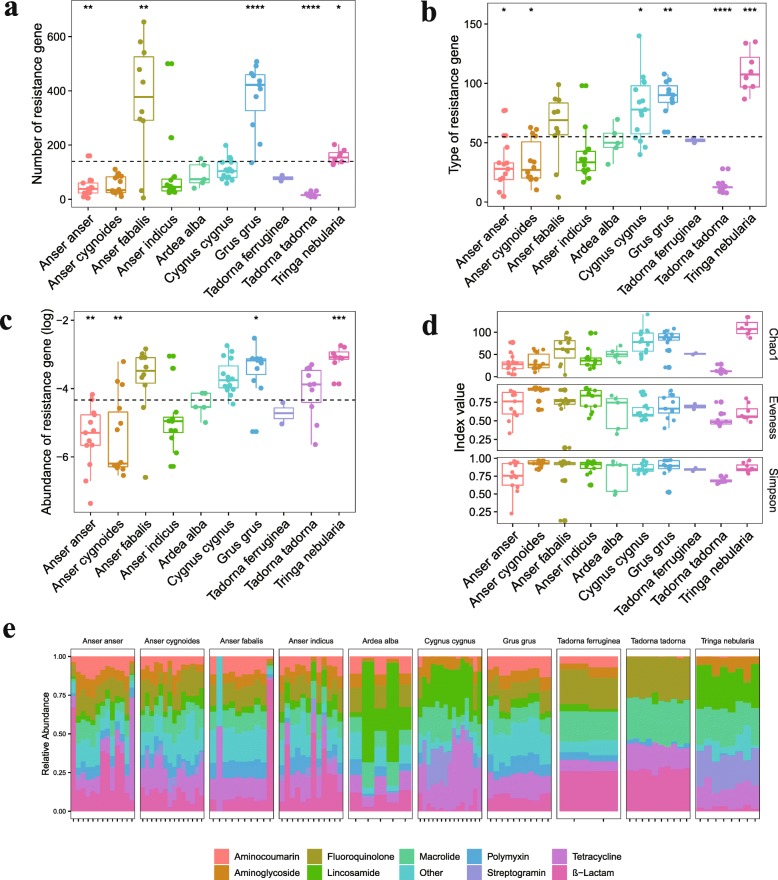
Comparison of resistance gene composition among bird species. **a** Number of resistance gene. **b** Type of resistance gene. **c** Abundance of resistance gene. **d** Resistome alpha diversity between bird species. **e** The relative abundance of antibiotic resistance gene type assigned to each major antibiotic class in a different population. Boxes denote the interquartile (IQR) between the first and third quartiles (25th and 75th percentiles, respectively) and the line inside denotes the median. Whiskers denote the lowest and highest values within 1.5 times and the IQR from the first and third quartiles, respectively. The asterisks on the top indicate *P < 0.05, **P < 0.01, and ***P < 0.001 (Mann–Whitney *U* test)

A total of 1030 ARGs were detected in the gut microbiomes, as shown in Fig. 3a. Each species carried a different number of ARGs, and Mann–Whitney tests suggest that *Anser fabalis*, *Grus grus*, and *Tringa nebularia* contained a higher number of ARGs than the average, with *Anser anser* and *Tadorna tadorna* lower than the average (Fig. 3a). The 1030 ARGs were grouped into 202 resistance gene types (Additional file 12: Table S4). The number of gene types in each species varied greatly, ranging from 53 to 165, with an average of 118, and one of the individuals contained the most types (140). We made further comparisons between the number of types among different bird species. More resistance gene types existed in *Cygnus cygnus*, *Grus grus*, and *Tringa nebularia*. Meanwhile, *Anser anser* and *Tadorna tadorna* harbored fewer types than the average (Fig. 3b). The relative abundance of individual ARGs was calculated using the original high-quality short reads based on sequence coverage. On average, the resistance genes accounted for 0.011% (*Anser indicus*), 0.066% (*Grus grus*), 0.047% (*Anser fabalis*), 0.042% (*Cygnus cygnus*), 0.019% (*Tadorna tadorna*), 0.004% (*Ardea alba*), 0.095% (*Tringa nebularia*), 0.007% (*Anser cygnoides*), 0.002% (*Tadorna ferruginea*), and 0.001% (*Anser anser*) of the total intestinal genes, respectively. Based on the widespread analysis of the abundance of ARGs in different bird species, *Grus grus* and *Tringa nebularia* had the most abundant ARGs (Fig. 3c). Several α-diversity indices for each species resistome were also calculated (Fig. 3d).

**Fig. 4:**
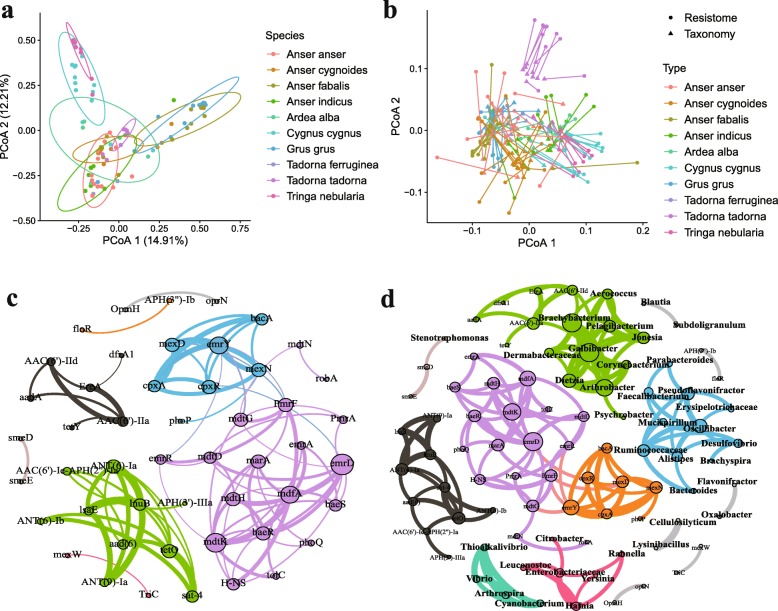
Resistome variation among different bird species. **a** ARG composition is influenced by host species. **b** ARG abundance were correlated with bacterial profiles in each species using Procrustes analyses. **c** Network analysis about the co-occurrence patterns among ARG subtypes. A connection represents a strong (Spearman’s correlation coefficient *ρ* > 0.85) and significant (*P* value < 0.01) correlation. The size of each node is proportional to the number of connections (i.e., degree). **d** Network analysis about the co-occurrence patterns between ARG subtypes and microbial taxa. A connection represents a strong (Spearman’s correlation coefficient *ρ* > 0.85) and significant (*P* value < 0.01) correlation. The size of each node is proportional to the number of connections(i.e., degree)

We then matched each resistance gene type to its corresponding antibiotic and summarized the relative abundance of types resistant to the same antibiotic. From the results, we concluded that the resistant gene types in the bird gut microbiota conferred resistance to almost all major antibiotic classes commonly administered for clinical and agricultural use. Among these antibiotics, tetracyclines, macrolide-lincosamide-streptogramin (MLS), and Beta-lactams contributed to most of the percentage of total resistance genes, and tetracycline resistance (TcR) genes were the most prevalent genes in all bird species (Fig. 3e). We next assessed the global similarity of ARG composition in each population based on PCoA. We found that bird species differed from each other in their resistomes, with species explaining the variation between samples (Fig. 4a), suggesting bird species contributed mostly to the ARG composition dissimilarity. The microbial origin of ARG was also analyzed. *Proteobacteria*, *Firmicutes*, *Bacteroidetes*, and *Fusobacteria* accounted for most of the phyla inferred from the resistance-conferring contigs. *Proteobacteria* was the dominant phylum in all species except for *Anser anser*, where *Fusobacteria* was the abundant phylum (Additional file 5: Figure S5).

We then identified a wide range of MGE sequences in bird metagenomic libraries, including phage-related integrases, putative transposases, type IV secretion systems, and reverse transcriptases. To further assess the potential mobility of ARGs, we identified a number of resistance genes located within the putative mobile elements in the same metagenomic contigs. Taking the *arnA* resistance gene as an example, it was encoded in a phage-related integrase. Similarly, efflux pumps (*mefA*, *emrA*, and *mdtG*) were also located within mobile elements, such as phage integrase and putative transposase. These pumps are recognized as intrinsic mechanisms to expel multiple antibiotics in multidrug-resistant bacteria. The MGEs associated with particular ARGs in bird gut microbiomes would promote the resistance dissemination via HGT among a diverse range of hosts.

### Relationship between bacterial phylogeny with ARG composition in migratory birds

A total of 21 ARG types were shared among different bird populations (Additional file 13: Table S5). Among these shared ARG types, the *Tet32* and *TEM-1* pose the highest risks to human health. The abundance of each ARG type in birds varied from population to population. Among the top 10 most abundant gene types (Additional file 14: Table S6), the *CRP* and *cpxR* types existed in eight bird populations (except for *Cygnus cygnus* and *Tringa nebularia*), whereas *Cygnus cygnus* and *Tringa nebularia* had more types in common (*ErmF*, *tet32*, *lnuC*, *ErmQ*, *APH (3′)-IIIa*, *msrE*, *tetQ*, and *ErmB*) than with other species.

To uncover the co-occurrence patterns of ARG types in bird populations, a network was established based on strong and significant correlations between different types that appeared in individuals (Spearman’s *r* > 0.85, *P* < 0.01). The network was comprised of 49 nodes and 93 edges, while the topological properties of the network were calculated to depict the complex co-occurrence pattern (Additional file 15: Table S7). A previous study indicates that a network with 0.634 modularity has a modular structure [20]. The network naturally fell into eight major modules; the largest three modules consisted of 34 of 49 total nodes (Fig. 4c). Each module showed dense connections between nodes, and consequently, the most densely connected node was defined as an indicator for co-occurrence among ARG types. From the results, we concluded that “*emrD*” is the hub of Module I, whereas “*emrY*” is the hub of Module II (Fig. 4c). In module III, there were two hubs: *ANT (6)-Ia* and *tetO*. We inferred that bird populations with similar bacterial compositions possibly contributed to the hubs and related co-occurring ARG types in each module.

As shown in the procrustes analysis results (Fig. 4b, *M*^2^ = 0.63, *P* < 0.001), the Bray–Curtis distance of microbial composition was not correlated with resistance types composition among individuals. Then, we selected strong correlation (Spearman’s *r* > 0.85, *P* < 0.01) and investigated the co-occurrence patterns between ARG types and microbial taxa using the network analysis approach. Topological properties and detailed information about the co-occurrence patterns were summarized (Additional file 16: Table S8). As shown in the network (Fig. 4d), three bacterial genera were speculated as possible ARG type hosts based on co-occurrence results; *Stenotrophomonas* mainly carried the multidrug resistance gene (*smeD*), whereas *Galbibacter* and *Brachybacterium* were the hosts of aminoglycoside resistance genes (*AAC (6′)-IIa* and *AAC (6′)-IId*). Our results are supported by the fact that the *smeD* resistance gene was first reported in *Stenotrophomonas maltophilia* [21].

### The difference between bird and its environmental microbiomes and resistomes

**Fig. 5:**
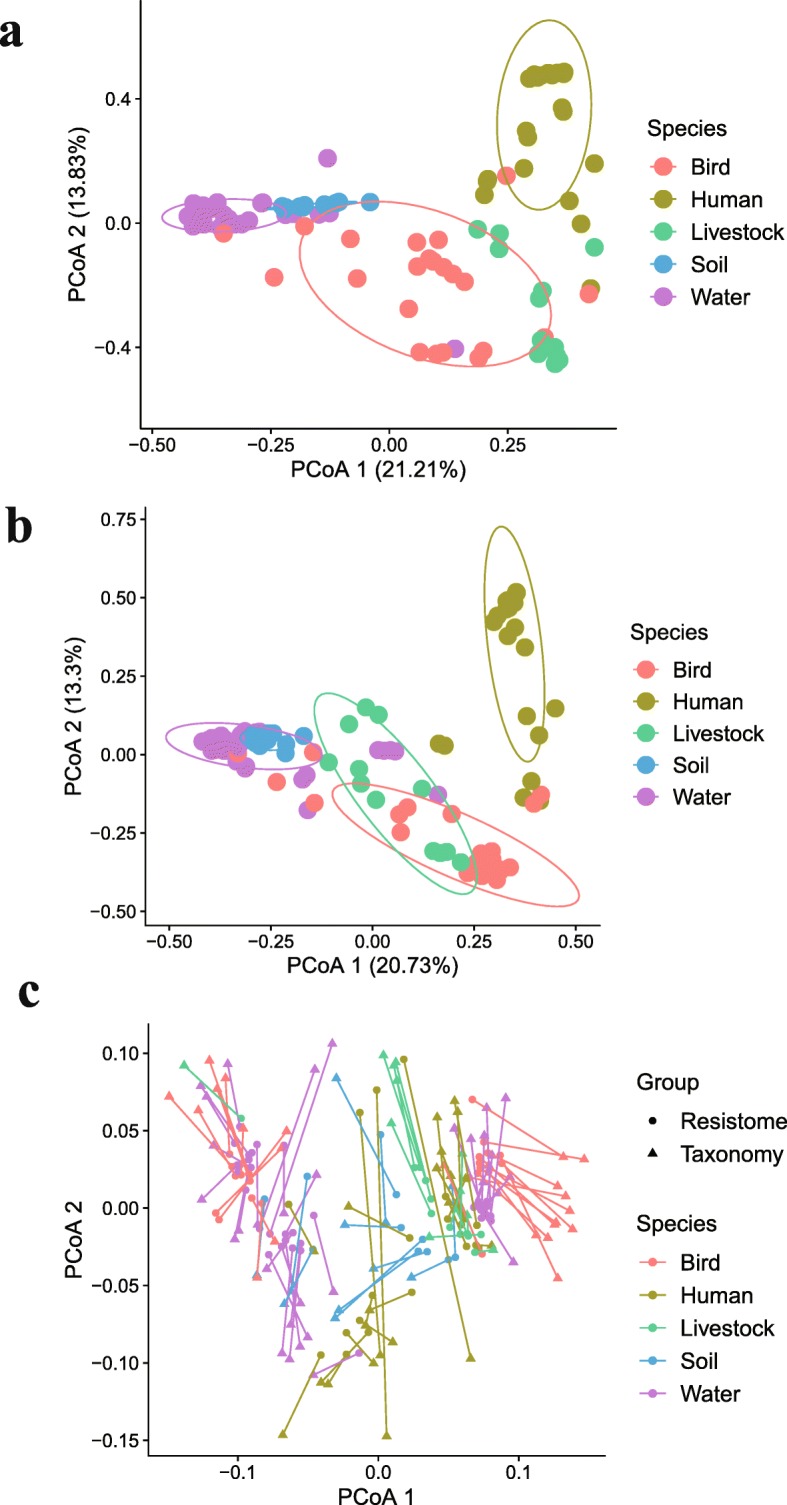
The dissimilarity of microbiome and resistome in the gut of bird and its associated environmental microbiota. **a** PCoA of Bray–Curtis distances between microbiota. **b** PCoA of Bray–Curtis distances between resistomes. **c** Procrustes analysis between taxonomic composition and resistome

We compared the metagenome sequencing data from wild bird feces with environmental samples from the Genga Lake (GGL) and Poyang Lake (PYL) area. The phylogenetic composition differed greatly across diverse ecological environments, and habitats contributed to 21.2% of microbiome variation (Fig. 5a). Results from resistome analysis also displayed a similar trend along ecological gradients, with habitat explaining the variation between samples (20.7%; Fig. 5b). To further characterize the resistome distribution pattern across habitats, procrustes analysis suggested that community structure shaped the ARG composition (*r* = 0.77, *P* < 0.01; Fig. 5c, Additional file 17: Table S9). *Proteobacteria* was the dominant reservoir of ARGs across diverse environments, whereas *Bacteroidetes* was the only major reservoir of ARGs in the human gut (Additional file 6: Figure S6).

**Fig. 6:**
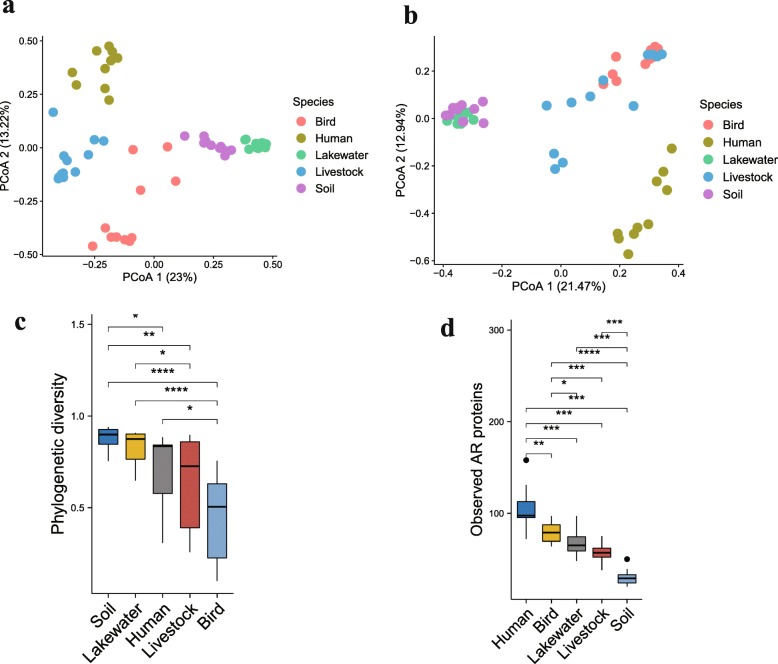
Bird fecal and environmental microbiota and resistome in Gengga Lake. **a** PCoA of Bray–Curtis distances between resistomes. **b** PCoA of Bray–Curtis distances between microbiota. **c** Simpson phylogenetic diversity. **d** Observed antibiotic resistance proteins. Boxes denote the interquartile (IQR) between the first and third quartiles (25th and 75th percentiles, respectively) and the line inside denotes the median. Whiskers denote the lowest and highest values within 1.5 times and the IQR from the first and third quartiles, respectively. The asterisks on the top indicate *P < 0.05, **P < 0.01, and ***P < 0.001, (Mann–Whitney *U* test)

Wild birds frequently interact with livestock effluent in the water. The excreta of livestock are often used as fertilizer without adequate treatment, potentially promoting antibiotic resistance exchange across various environments in GGL areas. Accordingly, the microbiomes and resistomes of wild bird were compared to their surroundings, including human feces, livestock feces, soils, and lake water. Bacterial abundance at the phylum level varied greatly across habitats (Additional file 7: Figure S7a, b). PCoA results indicated that the microbial structure of birds was different from soil and water along PC1 variation (Fig. 6a), while soil and water were more similar to each other. Livestock gut microbiota were close to human and distributed intermediately along PC1. The phylogenetic composition of the human gut microbiota displayed less heterogeneity than among the birds, soil, and water microbiota, potentially attributed to diverse and variable conditions in environmental samples. The resistomes of samples also separated from each other along ecological gradients (Fig. 6b). Birds were closer to livestock in ARG composition; they shared 86 antibiotic resistance proteins, including *ampC* beta-lactamase, *sul1*, and *sul2*. Although soil had the highest phylogenetic diversity (Fig. 6c), it harbored fewer ARGs per sample than other samples (Fig. 6d). In contrast, bird fecal microbiota contained the lowest phylogenetic diversity but had more ARGs per sample than both soil and water. In particular, the ARGs in birds were resistant to aminocoumarin, fluoroquinolone, and multiple antibiotics through antibiotic efflux mechanisms (Additional file 7: Figure S7 c, d).

**Fig. 7:**
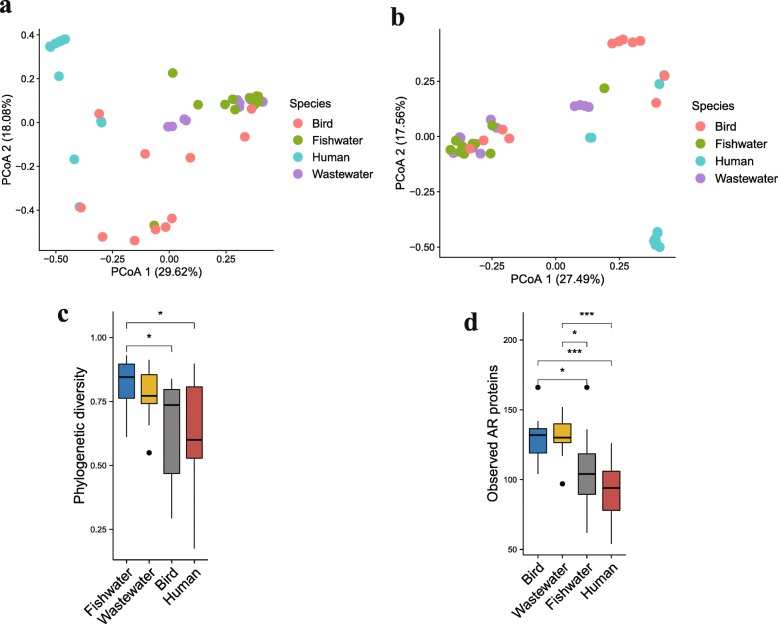
Bird fecal and environmental microbiota and resistome in Poyang Lake. **a** PCoA of Bray–Curtis distances between resistomes. **b** PCoA of Bray–Curtis distances between microbiota. **c** Simpon phylogenetic diversity. **d** Observed antibiotic resistance proteins. Boxes denote the interquartile (IQR) between the first and third quartiles (25th and 75th percentiles, respectively) and the line inside denotes the median. Whiskers denote the lowest and highest values within 1.5 times and the IQR from the first and third quartiles, respectively. The asterisks on the top indicate *P < 0.05, **P < 0.01, and ***P < 0.001, (Mann–Whitney *U* test)

The wastewater from households is directly discharged into Poyang Lake, potentially leading to contamination of environments with antibiotic residues and ARGs. We sampled water from households and fishery areas and compared their community structure and ARG composition to that from migratory birds. PCoA results showed that wastewater and fishwater were more similar in their microbial structure; they were abundant in *Proteobacteria* (Additional file 8: Figure S8a, b). The bird microbial structure was more heterogeneous than other samples, undergoing drastic changes in microbial similarity along PC1 and PC2 (Fig. 7a). Wastewater and fishwater also had similar resistomes. Some birds carried similar antibiotic resistance content compared with wastewater and fishwater, while others displayed distinct resistance genes (Fig. 7b). Although birds contained lower phylogenetic diversity (Fig. 7c), they had the highest ARGs per sample (Fig. 7d). Human feces harbored both the lowest phylogenetic diversity and ARGs per sample compared with environmental samples. Antibiotic target protection and antibiotic inactivation were more enriched in the human resistomes, whereas drug efflux mechanisms were more abundant in bird resistome (Additional file 8: Figure S8c, d).

We then identified 32 ARGs that were widespread in > 60% of samples across all GGL and PYL areas, including the sulfonamide-resistant dihydropteroate synthetase (DHPS) *Sul1*, *Sul2*, and *TEM-171* beta-lactamase. We further investigated MGEs in the metagenomic assemblies to assess transfer risk of antibiotic resistance across hosts. The *Sul1* gene with the transpoase and integrase was identified in human feces from the GGL area and in bird feces and fishwater from the PYL area, implying that the *Sul1* gene has the potential to be transmitted among diverse habitats. TEM β-lactamase (*TEM-171*) co-localized with transpoase was also found in bird and human feces from the PYL area. The *Sul2* gene was encoded in genetic contexts from bird samples, but none of the mobile proteins were identified in the same contig.

### Bird populations as reservoirs of novel β-lactamases

By using original Illumina reads to map the *mcr-1* gene, we found that approximately 30% of bird samples carried this gene. All of these *mcr-1*-positive samples were further confirmed by PCR amplification and Sanger sequencing. The results showed that 100% of the *Tadorna ferruginea* samples harbored *mcr-1*, while the lowest proportion was in *Grus grus* samples (~ 30%) (Table 1). Furthermore, the distribution of the *mcr-1* gene was not limited to one place: the samples from Hangzhou Bay contained the highest prevalence (~ 72%), followed by Qinghai Lake (~ 70%). The newly identified *mcr-1* gene variants (*mcr-1.2* to *mcr-1.10*) and novel colistin resistance determinants (*mcr-2* to *mcr-8*) were not found in our samples [22]. Though the *mcr-1* gene has been reported in wild birds [23, 24], the high prevalence of the *mcr-1* gene at the population level was surprising to us, because these bird species lived in remote habitats that are far from dense human populations and live poultry markets. We then searched for another well-known ARG, *NDM-1*, in our data, but it was not detected in our gene catalog or metagenomic reads. We then designed *NDM-1* gene primers to amplify it from samples through PCR for further confirmation [25]. The negative results suggest that the *NDM-1* resistance gene was not currently prevalent in the migratory bird population but should still be surveilled due to its potential public health threat.

**Table 1:** The prevalence of colistin resistance gene *mcr-1* in feces samples of birds

Species	Positive
*Grus grus* (*n* = 23)	33%
*Cygnus cygnus* (*n* = 20)	55%
*Tringa nebularia* (*n* = 22)	60%
*Anser indicus* (*n* = 21)	35%
*Anser fabalis* (*n* = 22)	37%
*Anser cygnoides* (*n* = 20)	35%
*Ardea alba* (*n* = 18)	85%
*Anser anser* (*n* = 20)	41%
*Tadorna ferruginea* (*n* = 8)	100%
*Tadorna tadorna* (*n* = 22)	65%

The fARGene software, based on probabilistic gene models, can directly identify ARG fragments from metagenomic reads and reconstruct them into full-length genes with high accuracy and efficiency [26]. Three hundred thirty-three unique β-lactamase genes were discovered in total from our metagenomic assemblies, encoding proteins with lengths ranging from 249 to 423 amino acids. Most of β-lactamase genes were grouped into class a (55.8%) and class b (26.7%), respectively (Table 2). When comparing with previously reported genes stored in the NCBI Genbank, there were 22 β-lactamase genes with 100% identity to previously annotated genes, including 11 from class a, seven from class c, and four from class d. We then retrieved the β-lactamase genes in available public metagenome datasets, including gut microbiomes from humans (1267 samples), pigs (287 samples), and chickens (628 samples). We found that 15 complete β-lactamase genes were present (100% amino acid identity and coverage) in human, pig, and chicken gut microbiomes. Additionally, two β-lactamase gene were mapped in all microbiome gene catalogs, one of which gene was first identified in *E. coli* isolated from animal food in January 2011 in Slovenija. One hundred fifty (45%) genes with amino acid sequence identity < 70% are believed to be novel, comprising class a (89), class b (51), and class d (10). Additionally, the highest relative number of novel β-lactamase genes was from class a among the four classes (89 of 186, 47.8%). One novel β-lactamase gene was mapped to the chicken gut microbiome gene catalog, and two novel β-lactamase genes were present in the human gut microbiome gene catalog with 100% amino acid identity.

**Table 2:** Predicted β-lactamase genes in metagenomic data

	Class_a	Class_b_3	Class_c	Class_d_1	Class_d_2
Id==100%	11	0	7	1	3
90%≤Id<100%	55	30	31	4	0
80%≤Id<90%	21	4	0	1	0
70%≤Id<80%	10	4	0	0	1
60%≤Id<70%	18	21	0	1	3
50%≤Id<60%	44	20	0	1	1
Id<50%	27	10	0	0	4
Total	186	89	38	8	12

## Discussion

In this study, the microbiomes and resistomes of 10 bird species, belonging to *Anseriformes* (seven species), *Ciconiiformes* (one species), *Charadriiformes* (one species), and *Gruiformes* (one species), were explored based on metagenomic sequencing. From our results, we concluded that the gut bacterial communities in migratory bird populations are distinct from each other, but the metabolism and function of the gut microbiomes are similar among them. ARGs in bird species differ from one another in number, type, and abundance, but TcR genes were the most prevalent group of ARGs in all species. Furthermore, bird species inhabiting the same environment tended to have similar composition of bacteria community and ARG composition. Compared with the microbiomes and resistomes in the environments (including human, poultry, soil and water), migratory birds harbored a lower phylogenetic diversity but had more ARGs. The *mcr-1* resistance gene was widespread among different birds, accounting for 50% of the total samples. Meanwhile, migratory birds also carried a large number of novel β-lactamases. In addition to our study for ARGs, migratory waterfowl species both carry the highly pathogenic avian influenza virus (AIV) H5N1, and they are the natural reservoir of all AIV subtypes, hence widely drawing public attention [27–29].

The abundance of the *Proteobacteria*, *Firmicutes*, *Bacteroidetes*, and *Actinobacteria* phyla accounts for a large proportion of the gut microbiota among bird species. However, each species varied significantly in the detailed composition at lower taxonomic levels. The dominant phylum *Firmicutes* consisted of class *Negativicutes* (28.7%) and *Clostridia* (26.2%) in *Cygcus cygcus* and class *Clostridia* (85.1%) in *Anser cygnoides*. Animal hosts can intake energy and absorb nutrients from the metabolism of carbohydrates, polysaccharides, sugars, and fatty acids, with the help of *Firmicutes* [30, 31]. The genus *Peptostreptococcacea_noname*, belonging to the *Peptostreptococcaceae* family, contributes a great abundance of *Clostridia*. However, the detailed roles performed in hosts by microbes from *Peptostreptococcaceae* family need to be further clarified. In the *Grus grus*, *Anser fabalis*, and *Tadorna tadorna* groups, the *Proteobacteria* was mainly composed of *Gammaproteobacteria* at the class levels in each species (69.7%, 69.3%, and 88.7%, respectively). On the genus level, *Pseudomonas* was the most abundant in *Grus grus* and *Anser fabalis*, whereas *Vibrio* was the most enriched in *Tadorna tadorna*. Previous research indicates that members of the *Pseudomonas* can produce bioactive substances to prevent pathogenic bacteria infection [32]. However, it still remains unclear for its role in the birds’ gut microbiomes, which is worthy of further investigation. Significant differences in the distribution of *Bacteroidetes* were also discovered, especially the enrichment of class *Bacteroidia* and genus *Bacteroides* in *Alba alba* and *Tringa nebularia* species. *Bacteroides* species have the capacity to decompose polysaccharides and the products released by *Bacteroides* are beneficial to the host [33]. Another significant difference is the higher abundance of *Actinobacteria* in *Anser indicus*, primarily due to the predominance of *Clavibacter* (5.1%) and *Actinoplanes* (5.0%). As most of the members from *Actinobacteria* are widespread in soil, water, or air, these bacteria in birds may have originated from the environment, implying that the bird gut microbiota is also influenced by bacterial transmission [34]. These bird species-specific genera may be indispensable for the adaptation of the host to extreme diets or environments. We further gained insight into the microbiological risks in migratory birds, where 59 opportunistic pathogens were detected. These notorious pathogens posed high risks to public health, and migratory birds appeared to be a potential source of infection. The development of high-throughput sequencing and bioinformatics analyses have made it easier to perform broad-scale surveys for all pathogens carried by migratory birds, without missing fastidious or unknown bacterial species, especially for pathogens that pose high risks to human.

Some ARGs are both resistant to antibiotics and likely to play other critical roles to play in the environment. For example, certain multidrug efflux pumps encoded in the chromosome provide host cells with the capacity to excrete chemical toxins and heavy metals [35]. The CRP and cpxR types were abundant in our metagenomic data. The CRP type is a regulator that modulates bacterial antibiotic resistance by repressing the coding of the multidrug efflux pump MdtEF. The CRP mutants can increase the resistance ability of bacterial strains [36]. CpxR acts as an activator to enhance the expression of the efflux pump and improve drug resistance, and it was previously identified previously as a regulator of the cell envelope stress response [37]. In addition, most antibiotics are secondary metabolic products of fungi and bacteria [38]. The microbes growing in the environment would be selected by various antibiotics and acquired antibiotic resistance capacity via HGT or other processes. Furthermore, other numerous environmental factors lead to antibiotic resistance in habitats, including radiation and pollution, but these mechanisms are yet unclear. ARGs were originally harbored by commensal bacteria but exchanged within the intestinal microflora or acquired by other pathogenic bacteria through HGT [4]. ARGs associated with MGEs can frequently switch hosts within a microbial community. If resistance genes are located on MGEs, they pose high risks to human health. In this regard, continued surveillance of resistance genes in migratory birds worldwide is urgently needed.

The emergence and spread of ARGs mainly results from abuse and misuse of antibiotics. Much research has been devoted to examining the direct correlation between antibiotic use and the degree of resistance [39]. Antibiotic resistance hotspots include sewage plants, pharmaceutical effluents, aquaculture wastewater, and livestock feces, which release ARBs into environments. Recently published work indicates that humans and domestic livestock can act as significant potential repositories of ARGs and most ARGs in pathogenic bacteria were derived from these sources [40]. ARBs have been frequently isolated from livestock farms, landfills, and wastewater treatment facilities. Numerous examples reveal that ARBs can spread from environments to migratory birds [41]. The tetracycline gene *Tet32* was first identified in the Clostridium-related human colonic anaerobe K10 and is widely distributed in the bovine rumen and in porcine feces [42]. *TEM-1*, conferring resistance to penicillin by hydrolyzing the β-lactam ring, has been frequently reported to be carried by various bacteria isolated from hospitals and clinics worldwide [43]. It is not possible to exclude the notion that the high prevalence of these two ARG types in bird populations was caused by HGT, and now birds are disseminators. A large quantity of antibiotics is discharged from hospitals and livestock farms into rivers, sediment, and soils [44]. Compared with conventional toxic chemical contaminants including heavy metals and organochlorine pesticides, antibiotics may persist in the environment for a long time [45], imposing selective pressure on the gut microbiota of birds. All of the above reasons may lead to abundant and diverse ARGs in migratory birds.

Carbapenemase-producing *Enterobacteriaceae* are notorious and colistin as the last-resort antibiotic is always used to treat its infections. Initially, Liu et al. have reported colistin resistance gene (*mcr-1*) was transmitted by plasmid and was prevalent in *Escherichia coli* and *Klebsiella pneumonia* from China [40]. Subsequently, the diverse members of *Enterobacteriaceae* with different hosts source was detected to carry this resistance gene in over 30 countries across every continent, would suggest the global distribution of *mcr-1* [46]. Alarmingly, a clinical isolate harbors both *NDM-5* and *mcr-1* [47] and is resistant to nearly all clinical antimicrobial agents. The isolates carrying *mcr-1* gene can circulate within hospital settings and then further transmit into pathogens, thereby leading to worrisome infections in high-risk patients.

Animal-originated isolates carry more percentage *mcr-1* than human clinical isolates, indicating its key role as sources and reservoirs of *mcr-1* [40]*.* The careless use of colistin in agriculture and poultry may contribute to high prevalence of *mcr-1* in bacteria from animals and its associated products [40], which should be banned [48, 49]. The earliest known occurrence of *mcr-1* gene in migratory birds was in a European herring gull in 2016 [24], the other report about recovered isolates from Kelp gull feces harbors *mcr-1* gene on plasmid [23]. Their results indicate wild birds carry the *mcr-1* gene but are of relatively low prevalence, whereas our findings reveal *mcr-1* gene are widespread in migratory birds. We infer that migratory birds acquire *mcr-1* gene from food-producing animals or from environments, such as river water. Clinical pathogenic bacteria with β-lactamases seriously threaten human health. Most β-lactamases were from environmental reservoirs and were capable of transmitting across diverse habitats with the help of MGEs. Some identical β-lactamases that are identified in bird, human, pig, and chicken microbiota may support this possibility. We inferred most of novel β-lactamase genes were functional due to the high accuracy of fARGene software (> 80% positive). We will further synthesize candidate novel genes and clone into the expression vector to confirm its functionality. Our findings provided the insight that migratory birds were regarded as important reservoirs for novel β-lactamase. Given the close bond between migratory birds and human, it is highly possible that such novel β-lactamase gene and its carrier organisms could be transferred to humans and posed potential threat to public health. To some extent, our results uncover that migratory birds may act as a significant carrier of *mcr-1* and β-lactamase genes, the next step we should do is to understand how migratory birds transmit it in different ecological niches and in the global continents.

## Conclusions

Bacterial composition in the gut varied greatly among migratory birds, but the microbiome metabolism and function were similar to each other. One thousand thirty different ARGs were grouped into 202 resistance types conferring resistance to tetracycline, aminoglycoside, β-lactam, sulphonamide. Microbial community structure not shapes the resistome in migratory birds. Compared with the microbiome and resistome in environments, migratory birds harbor a lower phylogenetic diversity, but have more antibiotic resistance proteins. The findings of this study should propel the global surveillance and risk assessment of ARGs in migratory birds, which will aid to prevent the transmission of ARGs into pathogenic microbes through HGT.

## Methods

### Stool sampling and data sources

Eight bird migratory routes are distributed across the world, three among them—the East Africa/West Asia Flyway, Central Flyway and East Asia/Australia Flyway—are through China [50]. The dominant species in these flyways comprise geese, ducks, and gulls that select aquatic habitats such as Qinghai Lake and Poyang Lake as stopovers during their migration. We chose critical stopovers as sampling sites to collect fresh droppings, when only a single species presents based on longer field surveys. To ensure all samples belong to different individuals, the stools are collected at least 1 m away from each other. In our study, we have taken a total of 196 fresh feces from 10 different waterfowl species during the 2016, across eight provinces in China (Additional file 1: Figure S1 and Additional file 9: Table S1). All samples were placed in sterile containers and transported to the laboratory via dry ice and then stored at − 80 °C until further processing. We have extracted DNA from samples within 2 days after sample collection in the laboratory.

The Genggahai Lake is located in rural area that stands on alpine meadow ~ 350 km southwest of Xining, Qinghai. Sheep and cattle were grazing on the grass lands nearby, drinking water directly from the river and excreting excrements in the water. A wide range of antibiotics were used in livestock to prevent high prevalence of various bacterial infectious agents. The manure of livestock as fertilizer was also pouring into fields to improve produce of crops. Poyang Lake was the largest freshwater lake in China and located in the northern part of Jiangxi Province. Humans catch and culture fish directly in lake. Antibiotics were used to improve the survival rate of fish fries. Most households were now linked to a district-wide sewage system that releases waste from communities to the nearby area of lake. Gengga Lake and Poyang Lake wetlands were located nearby multiple avian flyways; a large number of migratory waterfowls selected it as stopover or wintering land, including swan goose (*Anser cygnoides*), bean goose (*Anser fabalis*), great black-headed gulls (*Larus ichthyaetus*), brown-headed gulls (*Larus brunnicephalus*), bar-headed geese (*Anser indicus*), ruddy shelducks (*Tadorna ferruginea*), and great cormorants (*Phalacrocorax carbo*). They have the chance to contact directly with livestock or humans when foraging in the fields or swimming in the river. Bird feces were collected; meanwhile, we asked consent from participant to collect fecal samples, 15 Tibetan individuals in Gengga Lake area and 17 Han individuals in Poyang Lake area were to be sampled. The environmental samples, including soil, water, and animal feces from sheep and cows, were collected with the permission of the residents. In Gengga Lake area, we sampled 15 soil samples from areas adjacent to house, from cows and sheep coops, as well as mud from the river’s bank; we also collected 15 water samples from the Gengga Lake. In Poyang Lake area, 15 sewage wastewater samples nearby communities and the 15 water samples in fishing grounds were collected (Additional file 9: Table S1).

### DNA extraction and sequencing

MO BIO PowerSoil DNA isolation kit provided details about how to manipulate samples; we extracted DNA from soil according to its protocol [51]. For the stool samples, we extracted metagenomic DNA from approximately 300 mg feces as previously described in the HMP protocol, high temperature was beneficial to the cracking of bacteria, we heated samples at 65 °C for 10 min and 95 °C for 10 min during the operation. Water samples were filtered with sterile 0.22-μm filters, and metagenomic DNA was also extracted from the filter membranes using the MO BIO PowerSoil DNA isolation kit. The DNA was divided into two parts and stored in at − 20 °C until further use. The resulting concentrations and quality of sample DNA were examined via nanodrop instrument and agarose gel electrophoresis.

Based on protocols of NEXTflex Rapid DNA-Seq Kit (Illumina, 96 reactions), some critical process are indispensable to make preparations for sequencing including DNA fragmentation and adapter ligation. We followed the instructions of kit to construct library for sample with the insert size of 350 bp. We lastly sequenced the gut microbiomes of 103 representative samples from 10 bird species and 99 environmental samples (Additional file 10: Table S2). All libraries were then performed sequencing on Illumina Hiseq X10 platform with 2 × 150 bp paired reads (Novogene, Beijing, China).

### DNA sequence assembly and annotation

We processed Illumina metagenomic data through the MOCAT2 software package, which provided a pipeline for processing raw reads, assembly, gene prediction, extracting marker genes, and mapping reads to external databases [52]. To ensure data quality for downstream analysis, the raw reads generated from samples (40.8~147.3 were trimmed and filtered to remove low quality (Q ≤ 20) and short reads (length ≤ 45) using FastX Toolkit; SOAPAligner2 was used to align reads to the custom database chicken genome and the reads that match the database were removed (identity cutoff ≥ 90%; seed length, 30 bp; maximum mismatches, 10 bp) [53]. We obtained scaftigs (minimum length, 500 bp) by using SOAPDenovo v1.06 to assemble high-quality reads [54]. Revised assemblies were created by realigning reads to an existing assembly, then correcting for indels and base pair errors depending on mapping depth. Prediction of gene in scaftigs from each sample was processed based on MetaGeneMark program [55]. CD-HIT was used to cluster genes from each samples based on the parameters (identity > 95%, overlap > 90%) [56].

We aligned high-quality reads against the gene catalog using SOAPAligner2 (identity cutoff ≥ 95%) and calculated the corresponding relative abundance of each gene through two steps: (1) for each gene *i*, we used *N*_*i*_ represented the number of reads mapped to each gene and *L*_*i*_ represented the length of each gene, while the copy number of each gene (*C*_*i*_) was calculated *N*_*i*_ divided by *L*_*i*_ (*C*_*i*_ = *N*_*i*_/*L*_*i*_); (2) then, we estimated the relative abundance of each gene (*A*_*i*_) in each sample (*n* genes) based on the formula (*A*_*i*_ = *C*_*i*_/$\sum \limits_{i=1}^n{C}_i$).

EggNOG (v 4.5) database was regarded as an important and scalable public resource for functional annotation that based on Orthologous Groups (OGs) of proteins at different taxonomic levels [57]. KEGG databases (release 88.0) included a large of important information about the biological system, hence providing an avenue to understand the microbiome functions in hosts from metagenomic datasets [58]. DIAMOND, an open-source algorithm, can align DNA and protein sequences quickly to corresponding database [59]. Here, we mapped the amino acid sequences of gene catalog into the proteins in eggNOG and KEGG databases using DIAMOND program with default parameters (*e* value ≤ 1e− 5), then selected the highest-scoring annotated hit (HSP > 60 bits). We summed the relative abundance of genes in same functional level as relative abundance of each functional category.

The Comprehensive Antibiotic Resistance Database (CARD) contained a great deal of known ARGs and its associated resistant antibiotics. Subsequently, we mapped sequence of the metagenomic gene catalog to CARD database to predict resistomes [60]. To ensure the accuracy of ARGs, we selected 80% identity cut off as the search criteria [61–63].

Bacterial, archaeal, viral, and eukaryotic are distinct from each other in genomes, MetaPhlAn2 can distinguish hosts based on 1 M unique marker genes that detected in those reference genomes; hence, it is a significant software to resolve the microbial communities structure in metagenomic data. In order to understand taxonomic information of our samples, the clean reads were compared against precompiled marker catalogs from available microbial genomes through nucleotide aligner Bowtie2 [64].

### Public data used

In 2014, scientists analyzed together the 249 new metagenomic data about human feces and 1018 samples sequenced before based on the MOCAT pipeline, they lastly obtained 9,879,896 genes and established a high-quality human gut microbiome gene catalog. The 1267 fecal samples were from Danish, Spanish, Chinese, and Americans [65]. The GigaScience database stored the nucleotide sequences and amino acid sequences of gene catalogs, gene profile, genus profile, KO profile, KEGG profile of the 287 pig gut metagenome, all the sets of data were downloaded for further analysis about ARGs [8]. The recent research revealed the ARGs prevalence in live poultry markets [9]; the metagenomic data were also included and compared it with the migratory birds as well.

### Statistical analysis and network analysis

Averages and standard deviations were computed using the base function in R 3.4.1 [66]. Venn diagrams were drawn with Venn Diagram package, while heatmaps were generated with the pheatmap package by R 3.4.1 [67]. One-way analysis of variation (ANOVA) was performed through ggpubr and ggplot2 packages (*p* < 0.05) [68]. The α-diversity and Shannon index on the gene, genus, and KO profile in each sample was calculated to evaluate the gene, genus, and KO diversities by R 3.4.1. We made principal coordinates analysis based on Bray–Curtis distance and then compared the dissimilarity of bacterial composition and ARGs profiles between species on R 3.4.1 in the vegan package [69]. To reveal relationship between microbial composition and resistome, we calculated the pairwise Spearman’s rank correlation, removed the correlation in which coefficient is below 0.85 and the *P* value is above 0.01, and adjusted the *P* value to avoid false positives using the FDR method [70]. Network analysis and visualization were conducted in R platform with the igraph package [71].

## Supplementary information

**Additional file 1: Figure S1.** The Map of Sampling Sites in China. The sampling sites are located in blue small flags on map including 8 provinces in China. The map in the lower right corner indicates islands in the South China Sea.

**Additional file 2: Figure S2.** The classes are presented in four major dominant phyla. (a) Proteobacteria, (b) Firmicutes, (c) Bacteroidetes.

**Additional file 3: Figure S3.** Alpha-diversity of bacterial communities in all bird samples. (a) Observed genera, (b) Shannon index.

**Additional file 4: Figure S4.** The functional annotation of predicted non-redundant gene catalog based on functional database. (a) KEGG annontation, and (b) eggNOG annotation.

**Additional file 5: Figure S5.** Phylogenetic origins of antibiotic resistance genes among bird population.

**Additional file 6: Figure S6.** Phylogenetic origins of antibiotic resistance genes across diverse habitats.

**Additional file 7: Figure S7.** bird fecal and environmental microbiota and resistomes in Gengga Lake. (a) Relative abundances of microbial phyla, (b) Relative abundances of microbial family, (c) Absolute abundances of antibiotic resistance categories, (d) Absolute abundances of antibiotic resistance mechnism. Boxes denote the interquartile (IQR) between the first and third quartiles (25th and 75th percentiles, respectively) and the line inside denotes the median. Whiskers denote the lowest and highest values within 1.5 times and the IQR from the first and third quartiles, respectively.

**Additional file 8: Figure S8.** bird fecal and environmental microbiota and resistomes in Poyang Lake. (a) Relative abundances of microbial phyla, (b) Relative abundances of microbial family, (c) Absolute abundances of antibiotic resistance categories, (d) Absolute abundances of antibiotic resistance mechnism. Boxes denote the interquartile (IQR) between the first and third quartiles (25th and 75th percentiles, respectively) and the line inside denotes the median. Whiskers denote the lowest and highest values within 1.5 times and the IQR from the first and third quartiles, respectively.

**Additional file 9: Table S1.** The detailed information of sample collection.

**Additional file 10: Table S2.** Detailed information of sequencing depth and antibiotic resistance gene number in 100 samples from different bird species.

**Additional file 11: Table S3.** Bacterial taxonomic classifications via Metaphlan.

**Additional file 12: Table S4.** ARGs abundance in 100 samples from different bird species.

**Additional file 13: Table S5.** ARG types present in all bird species.

**Additional file 14: Table S6.** The 10 most abundant ARG types present in each bird species.

**Additional file 15: Table S7.** Network properties on co-occurrence patterns of ARG types in bird populations.

**Additional file 16: Table S8.** Network properties on co-occurrence patterns between ARG types and micriobial taxa in bird populations.

**Additional file 17: Table S9.** Detailed information of sequencing depth and antibiotic resistance gene number in 99 samples from bird and its associated environments.

## Data Availability

All data generated during this study is available at the Sequence Read Archive (SRA) under BioProject number PRJNA556790 and PRJNA563508.
