# Supplementary figures and images for: Metagenomic analysis reveals the microbiome and resistome in migratory birds
Source: Microbiome. 2020 Mar 2;8:26. doi: 10.1186/s40168-019-0781-8 (PMC7053137; doi:10.1186/s40168-019-0781-8)

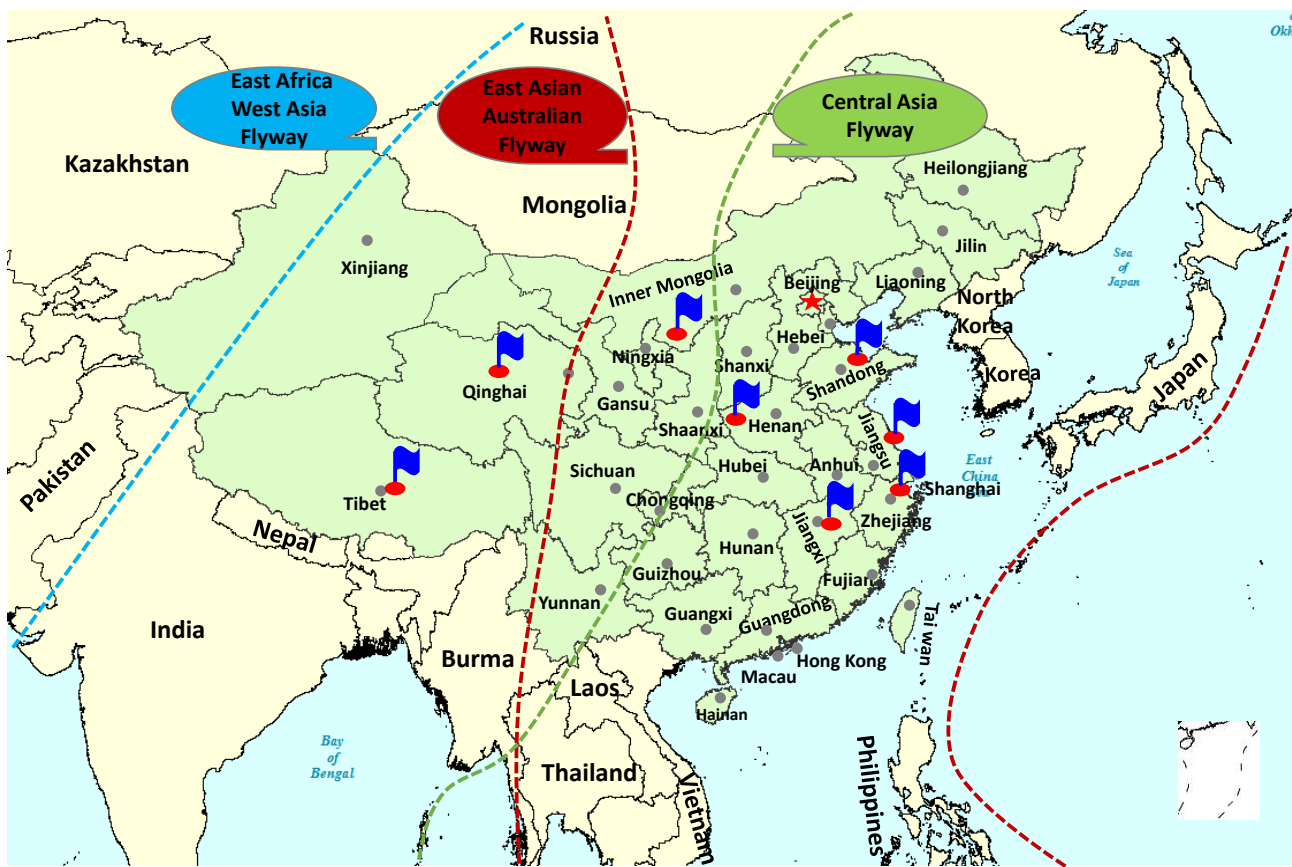

Supplement: Supplementary file 2 — Additional file 1: Figure S1. The Map of Sampling Sites in China. The sampling sites are located in blue small flags on map including 8 provinces in China. The map in the lower right corner indicates islands in the South China Sea. [file 40168_2019_781_MOESM1_ESM.pdf]

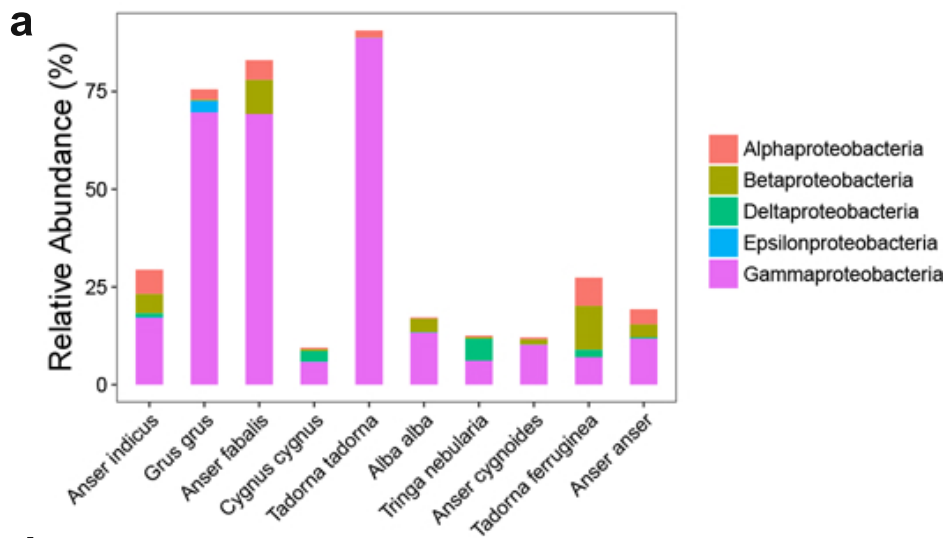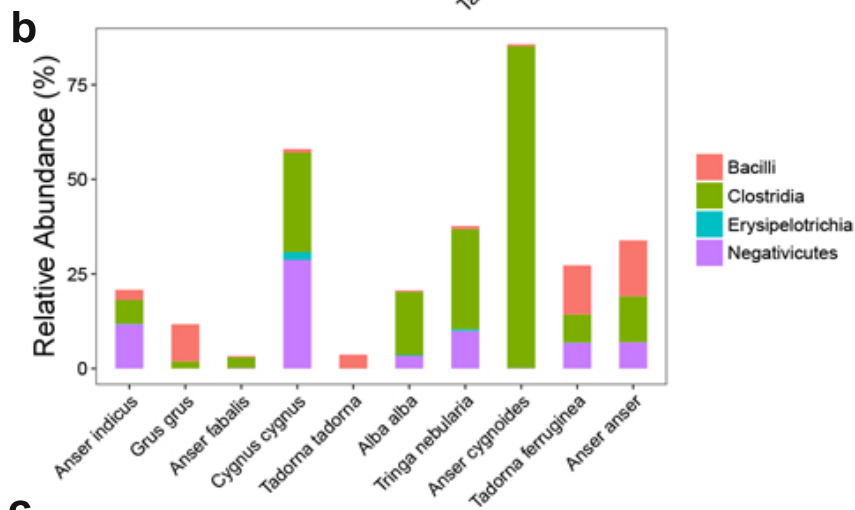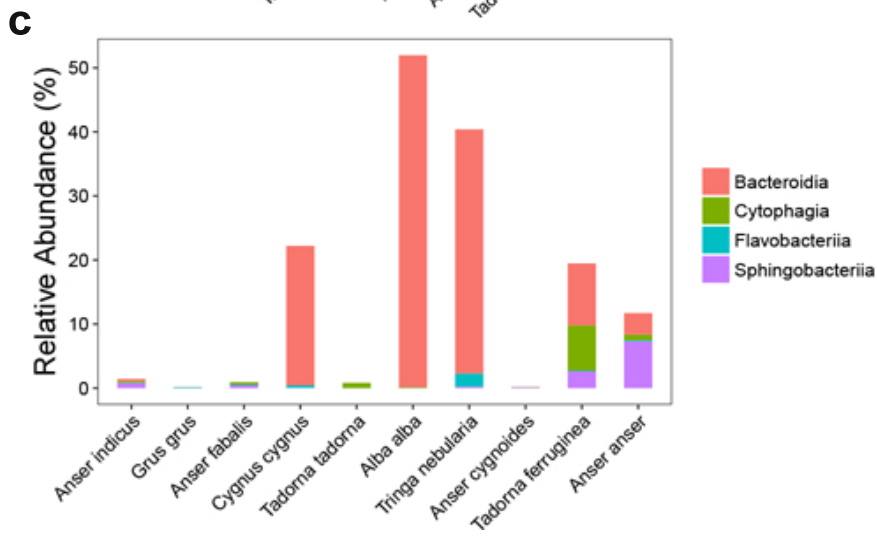

Supplement: Supplementary file 3 — Additional file 2: Figure S2. The classes are presented in four major dominant phyla. (a) Proteobacteria, (b) Firmicutes, (c) Bacteroidetes. [file 40168_2019_781_MOESM2_ESM.pdf]

**a**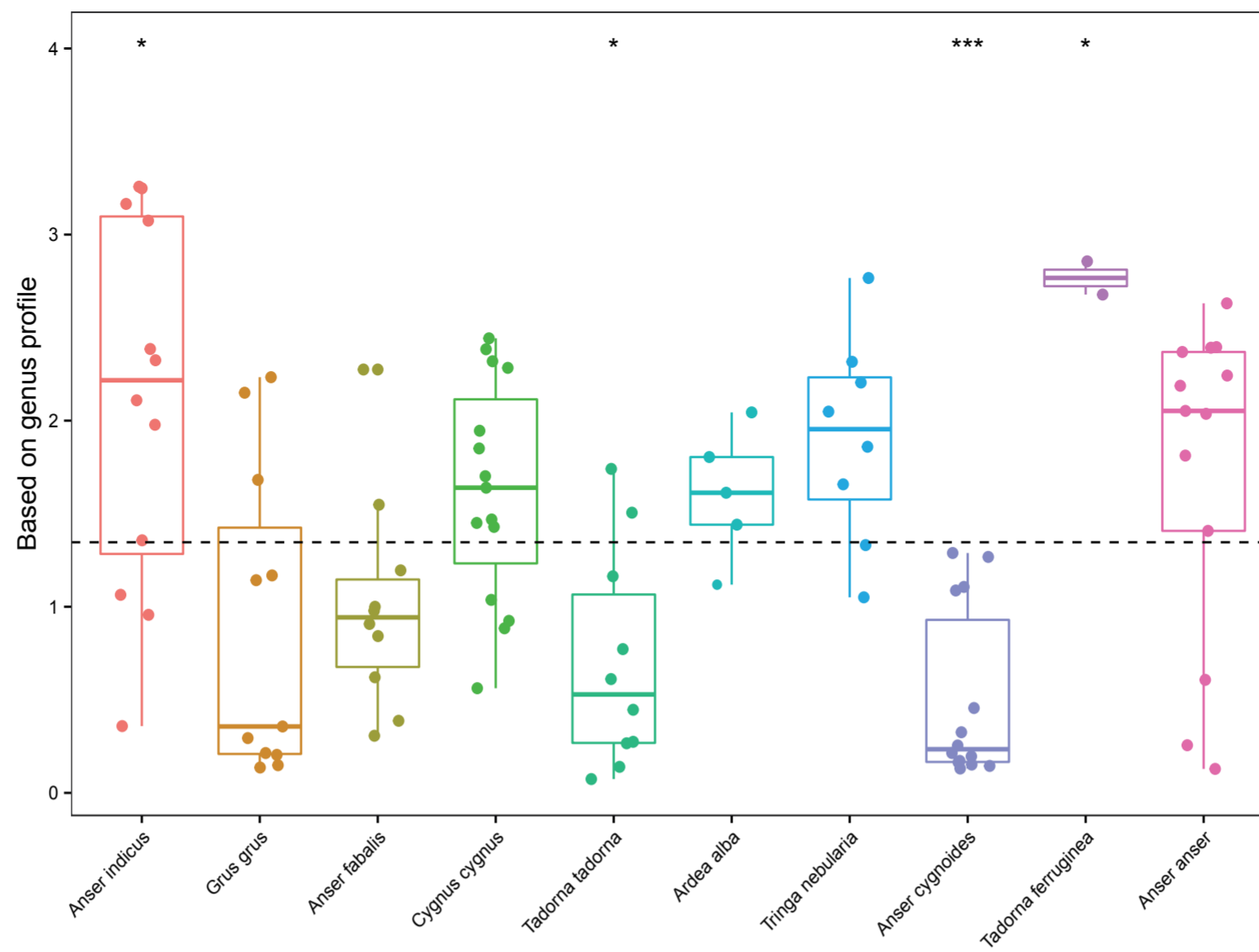**b**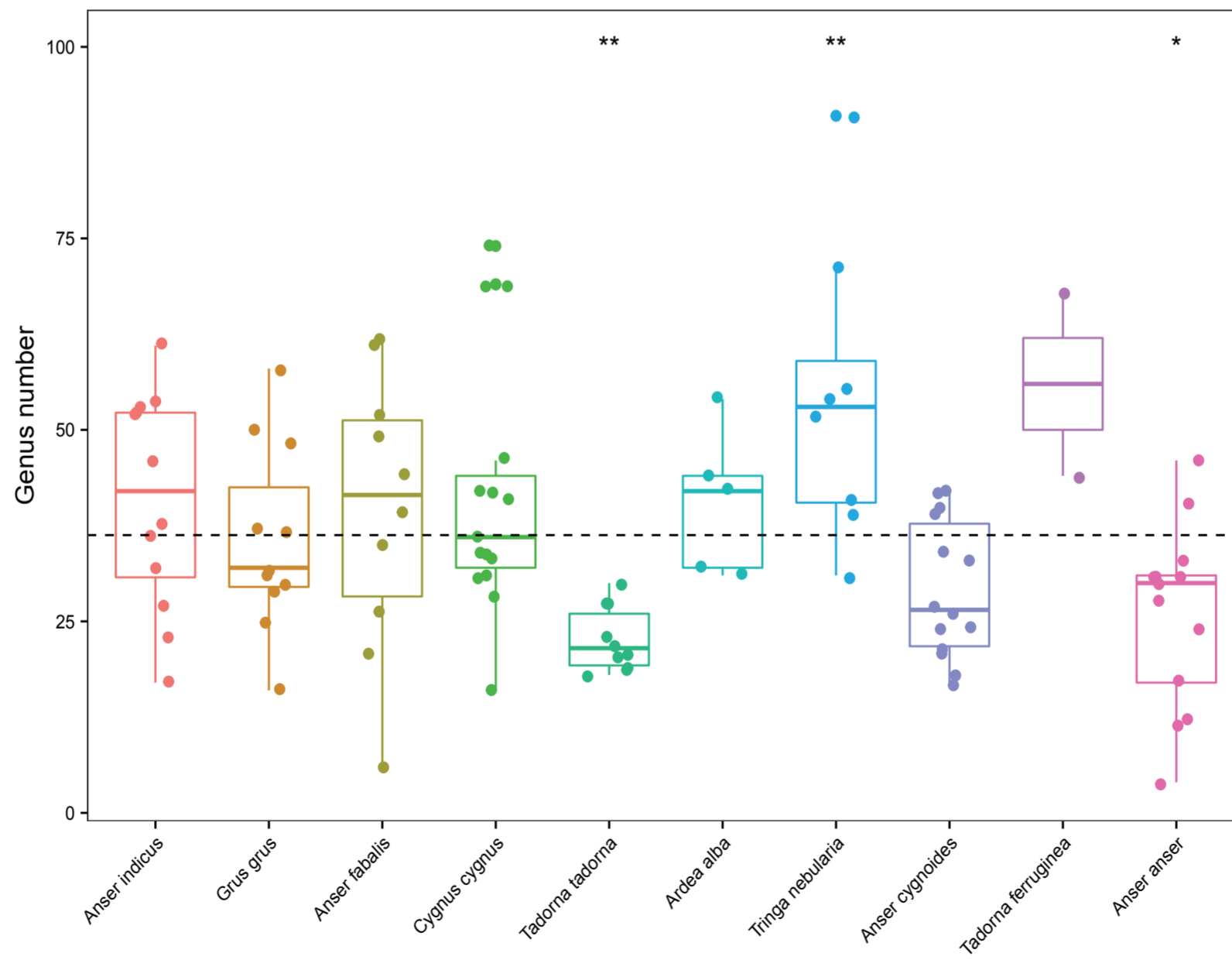

Supplement: Supplementary file 4 — Additional file 3: Figure S3. Alpha-diversity of bacterial communities in all bird samples. (a) Observed genera, (b) Shannon index. [file 40168_2019_781_MOESM3_ESM.pdf]

a

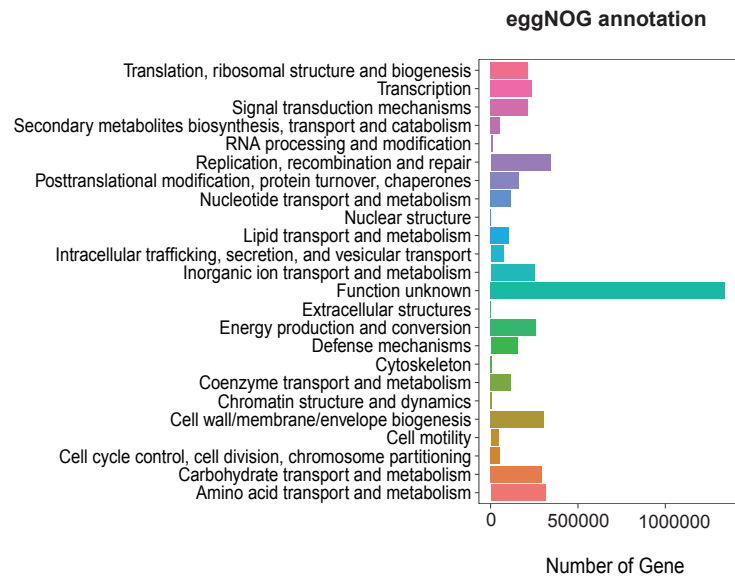

b

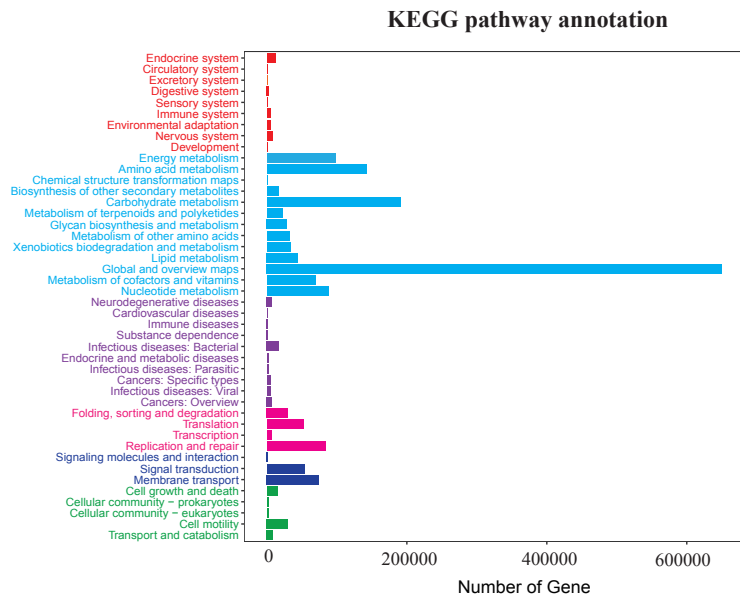

Supplement: Supplementary file 5 — Additional file 4: Figure S4. The functional annotation of predicted non-redundant gene catalog based on functional database. (a) KEGG annontation, and (b) eggNOG annotation. [file 40168_2019_781_MOESM4_ESM.pdf]

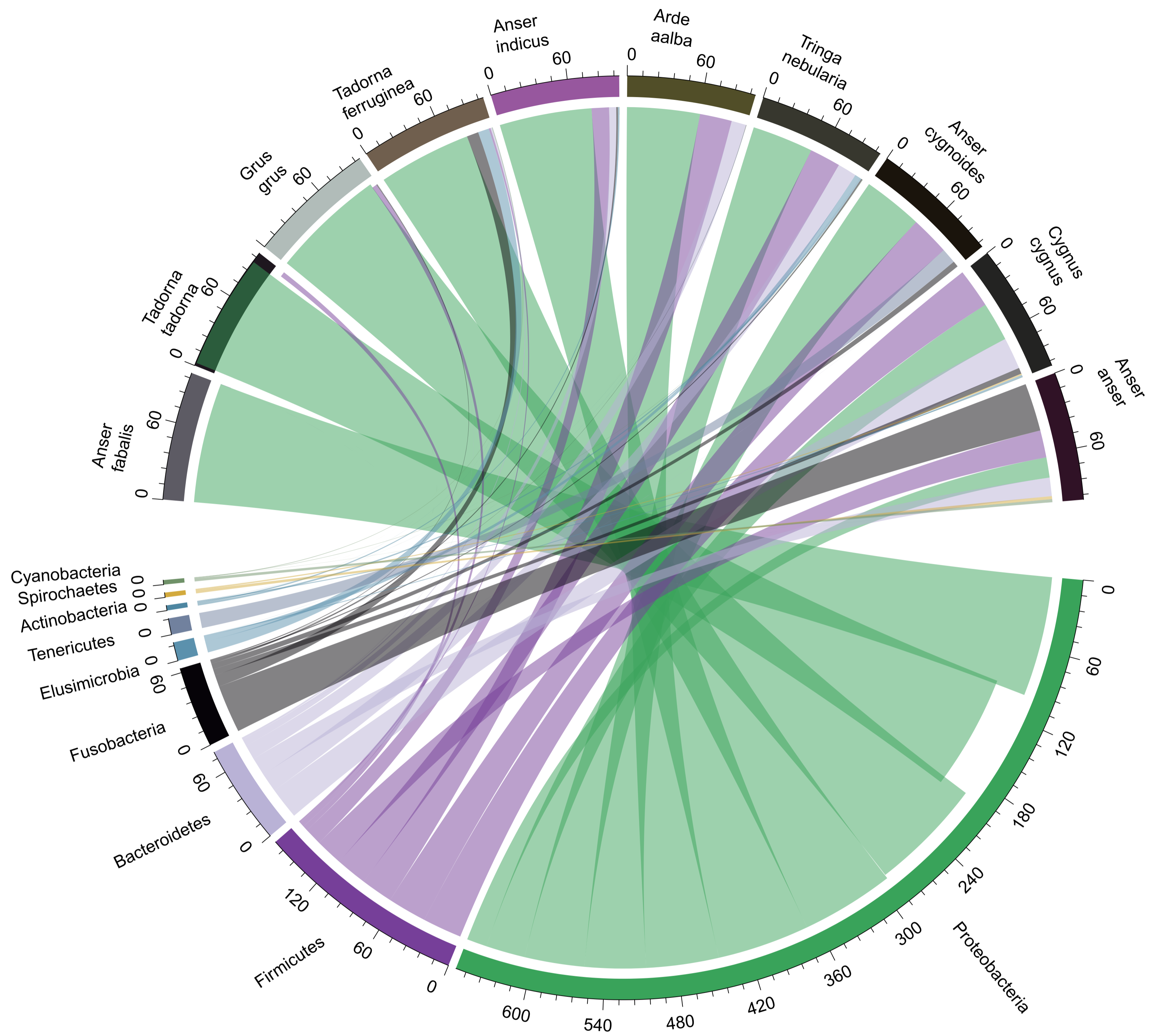

Supplement: Supplementary file 6 — Additional file 5: Figure S5. Phylogenetic origins of antibiotic resistance genes among bird population. [file 40168_2019_781_MOESM5_ESM.pdf]

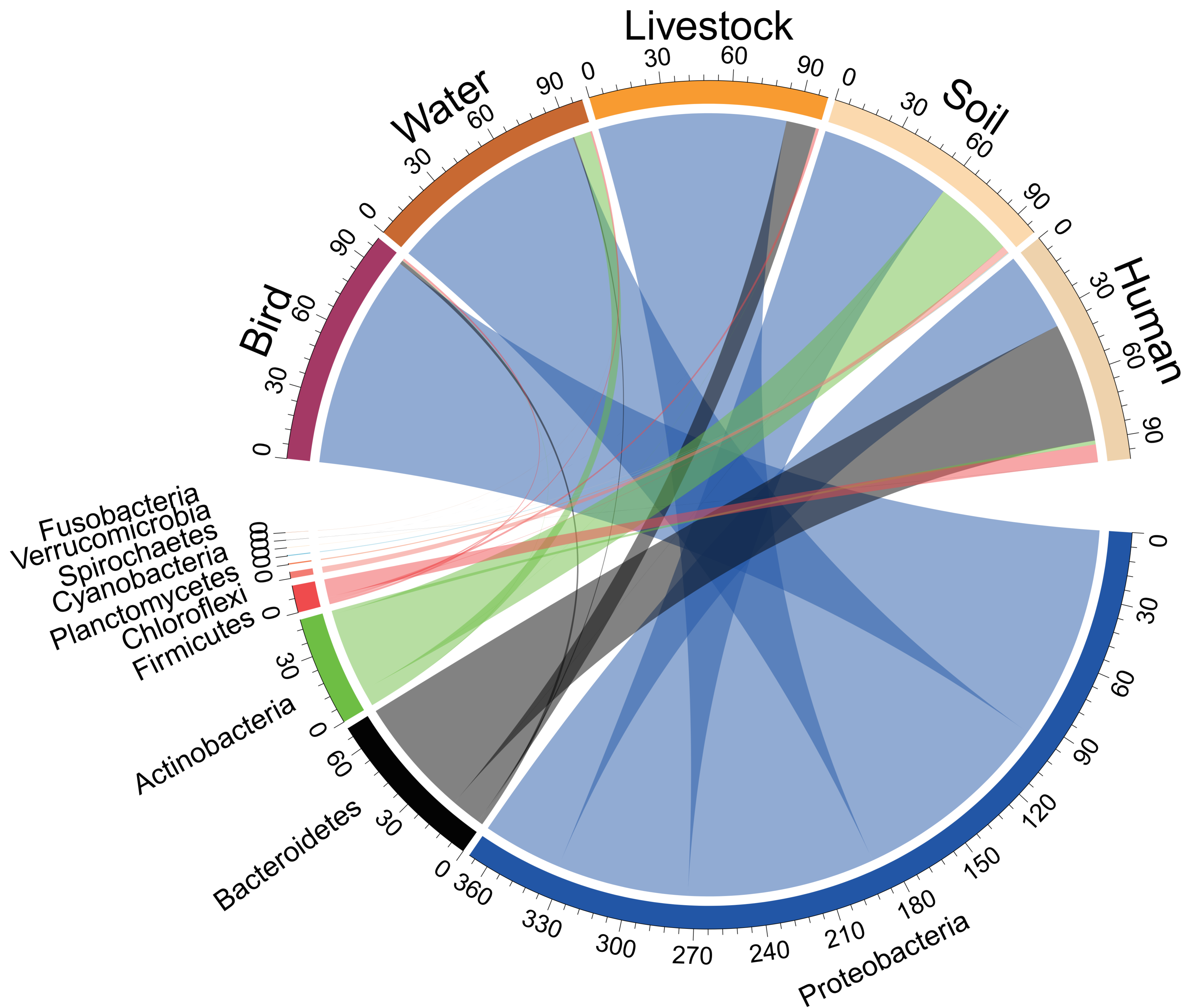

Supplement: Supplementary file 7 — Additional file 6: Figure S6. Phylogenetic origins of antibiotic resistance genes across diverse habitats. [file 40168_2019_781_MOESM6_ESM.pdf]

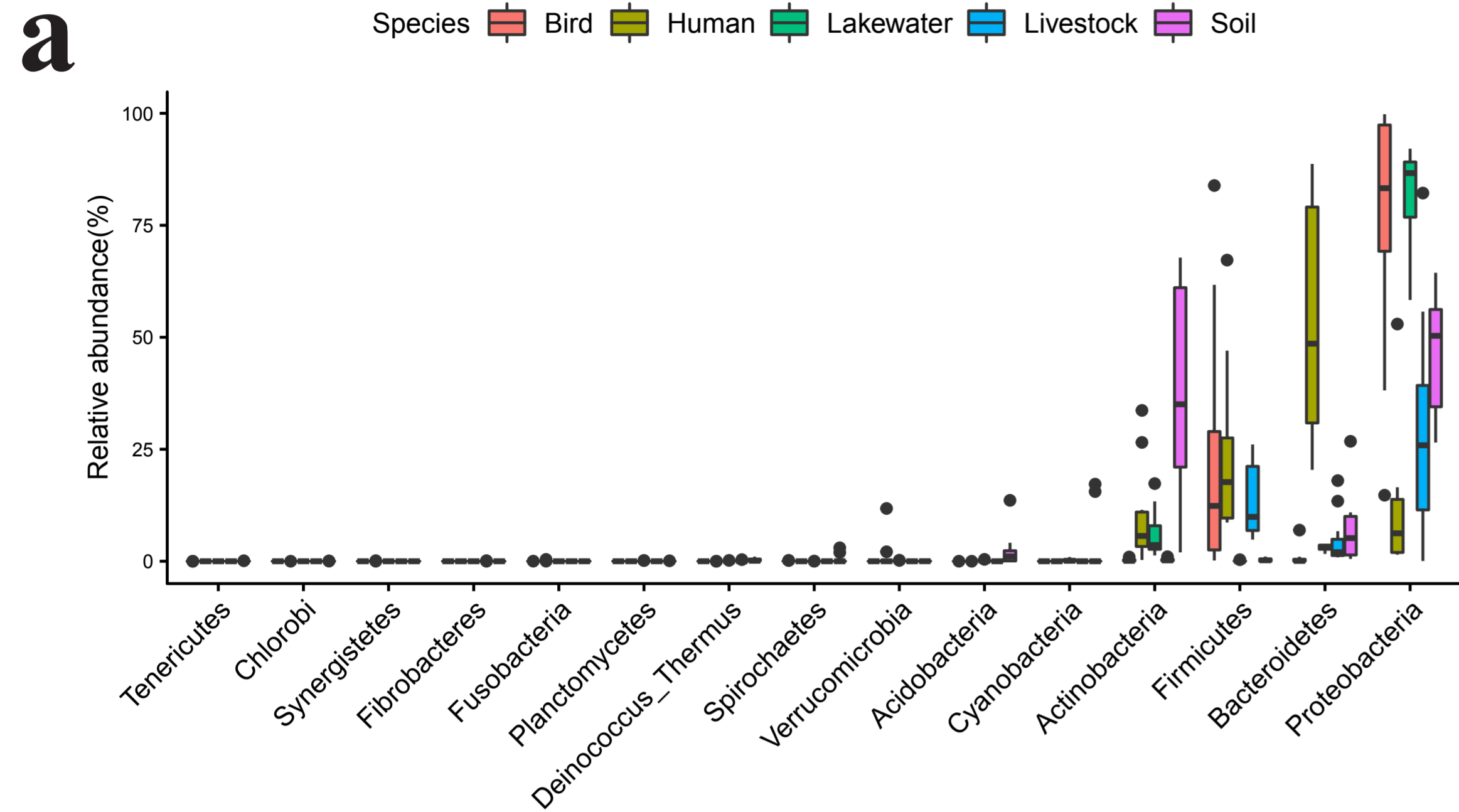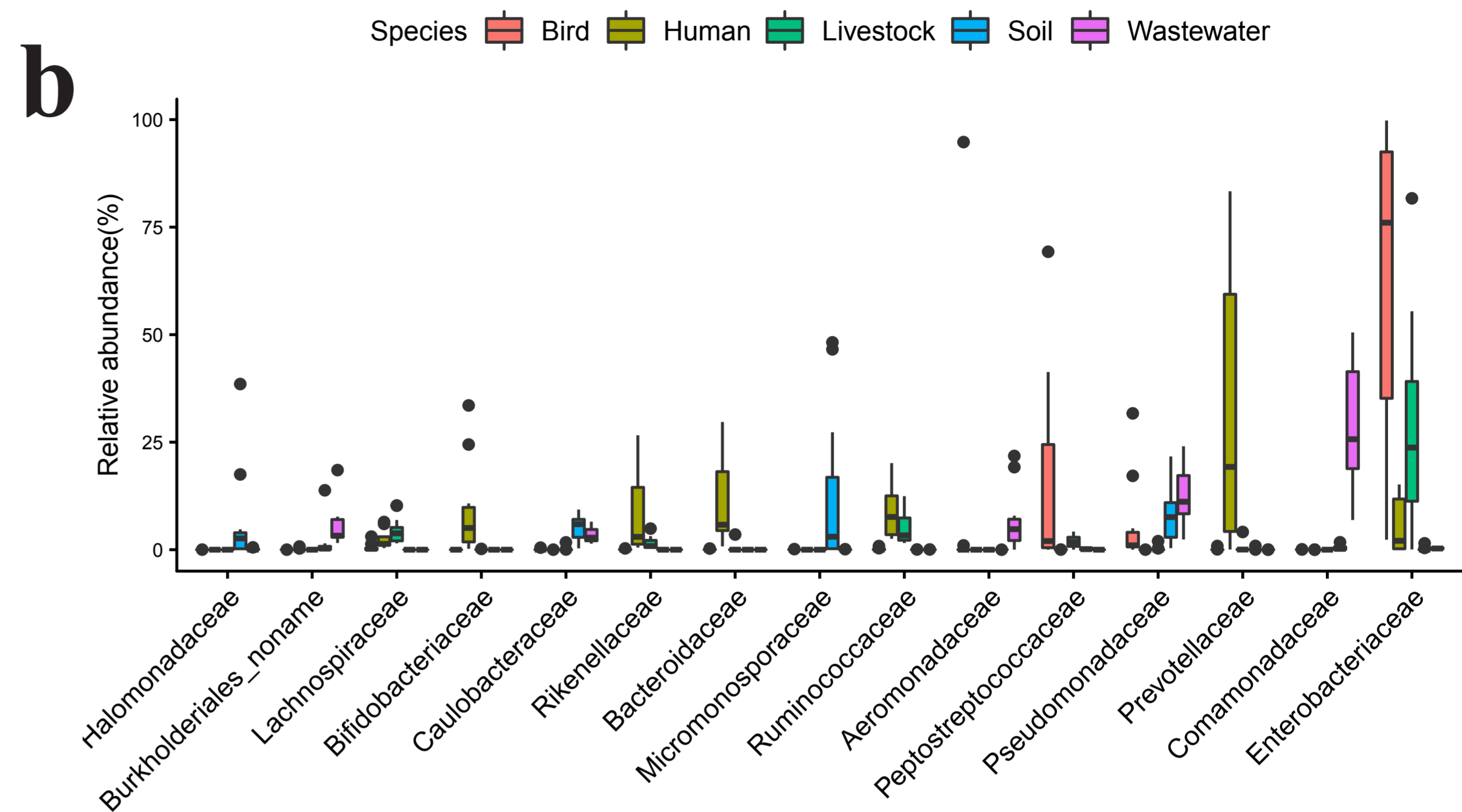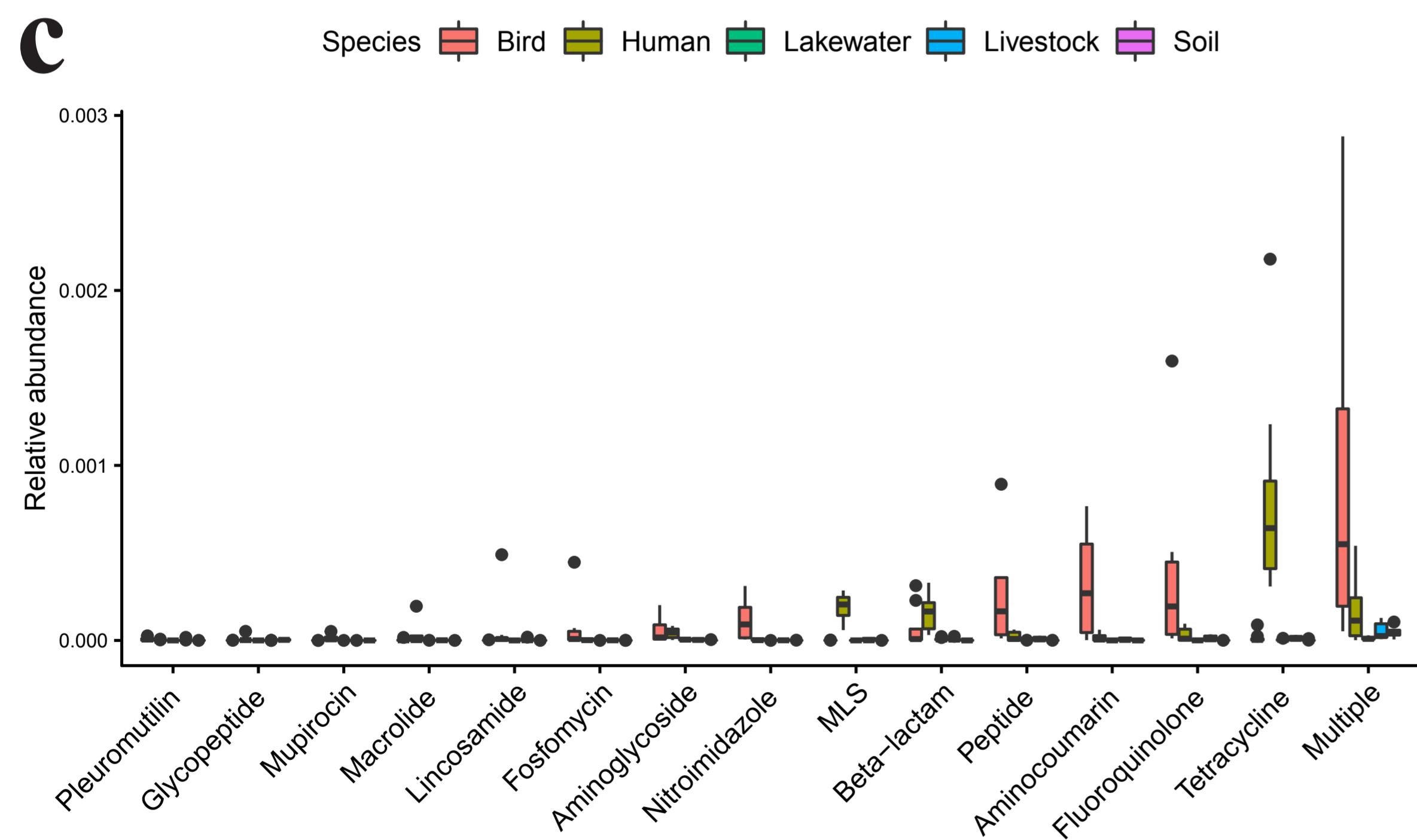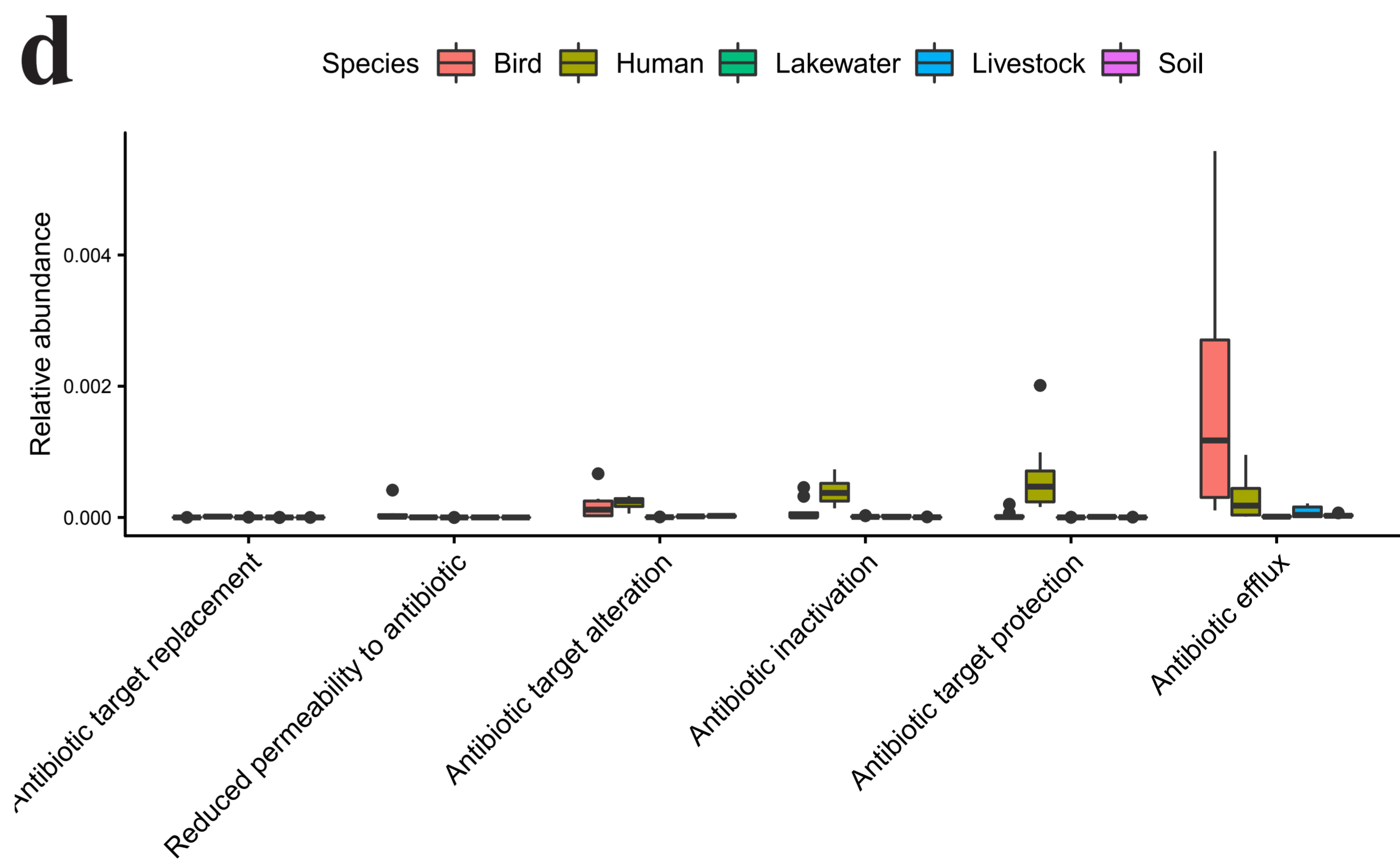

Supplement: Supplementary file 8 — Additional file 7: Figure S7. bird fecal and environmental microbiota and resistomes in Gengga Lake. (a) Relative abundances of microbial phyla, (b) Relative abundances of microbial family, (c) Absolute abundances of antibiotic resistance categories, (d) Absolute abundances of antibiotic resistance mechnism. Boxes denote the interquartile (IQR) between the first and third quartiles (25th and 75th percentiles, respectively) and the line inside denotes the median. Whiskers denote the lowest and highest values within 1.5 times and the IQR from the first and third quartiles, respectively. [file 40168_2019_781_MOESM7_ESM.pdf]

**a**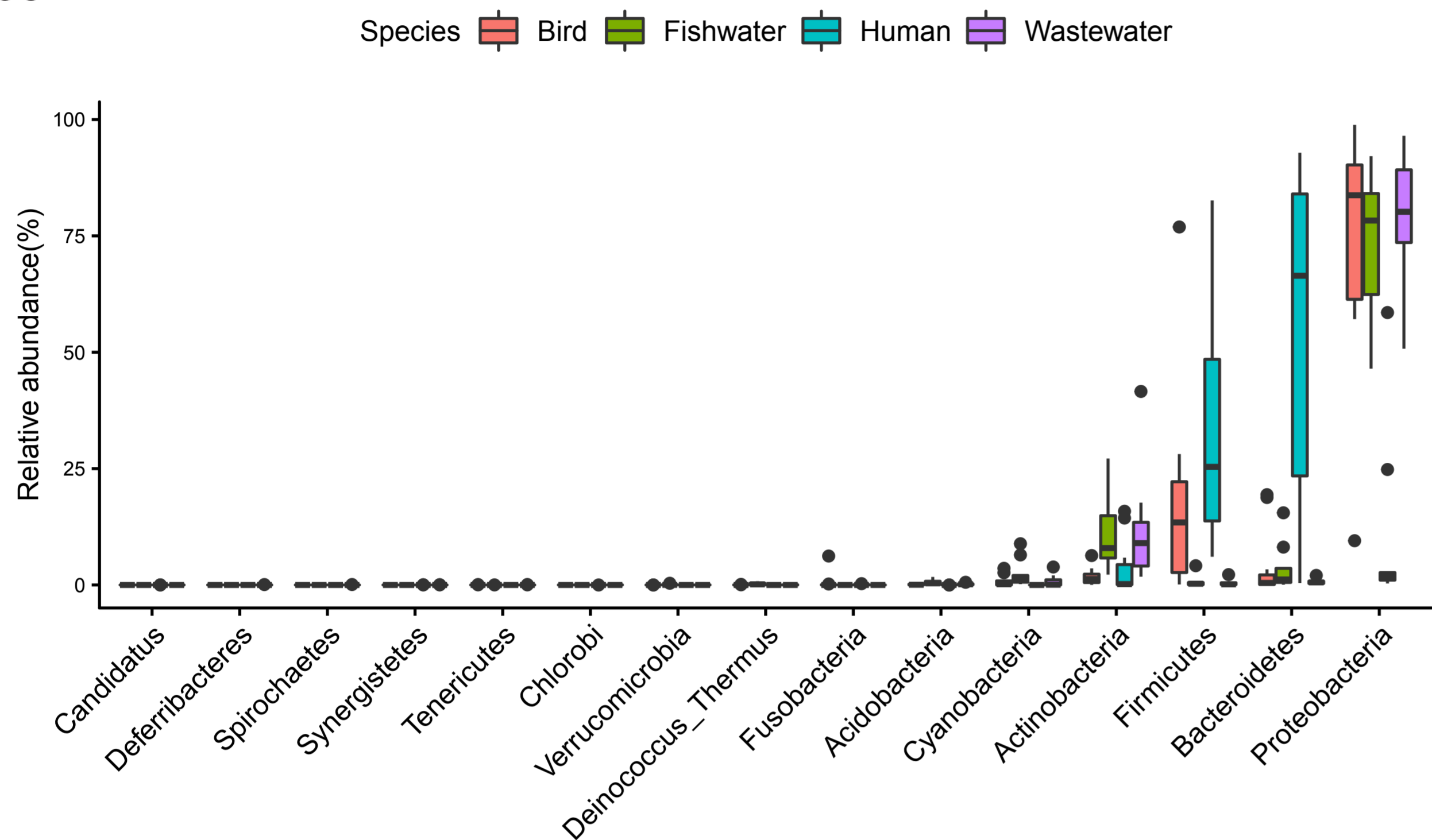**b**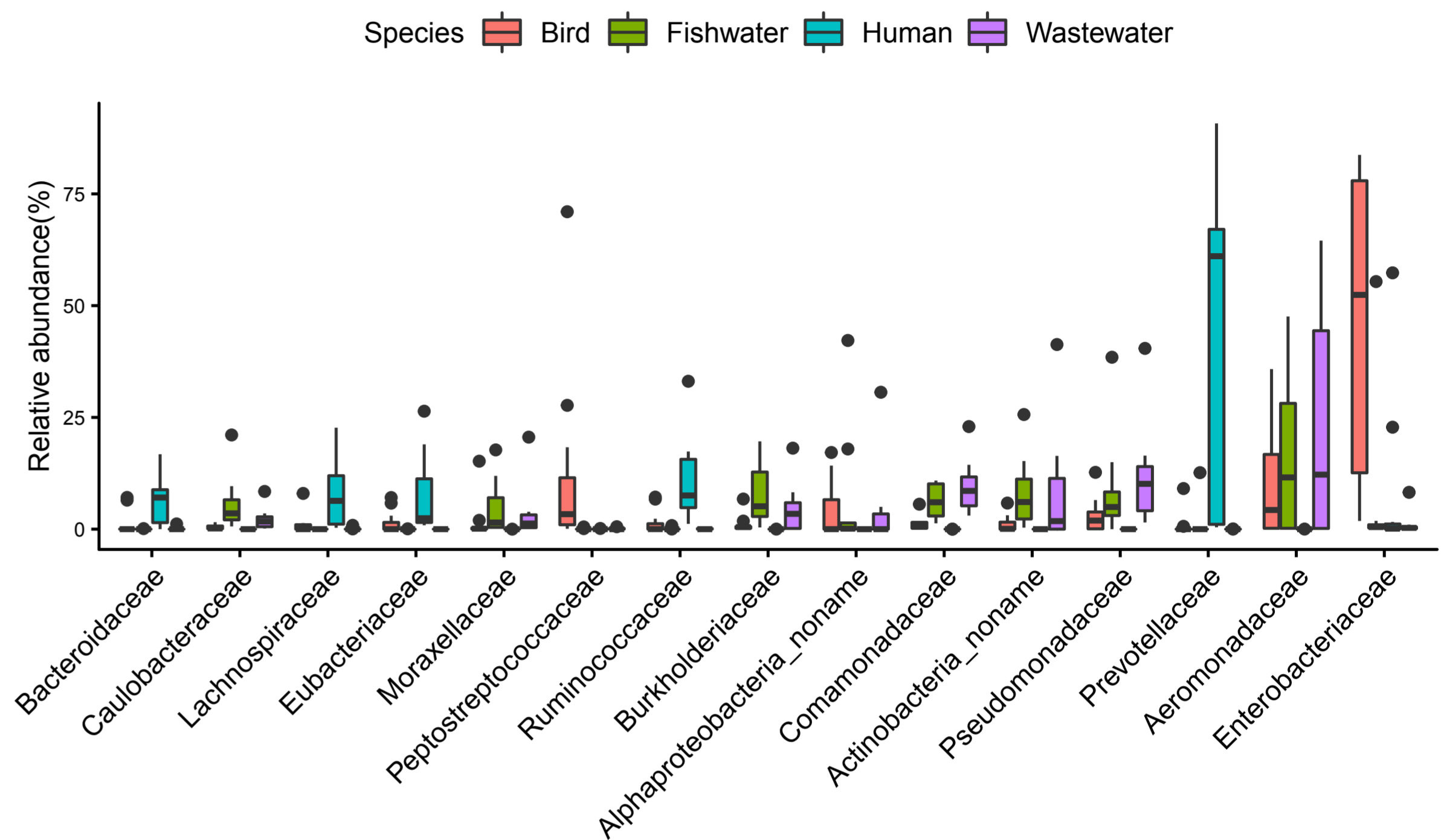**c**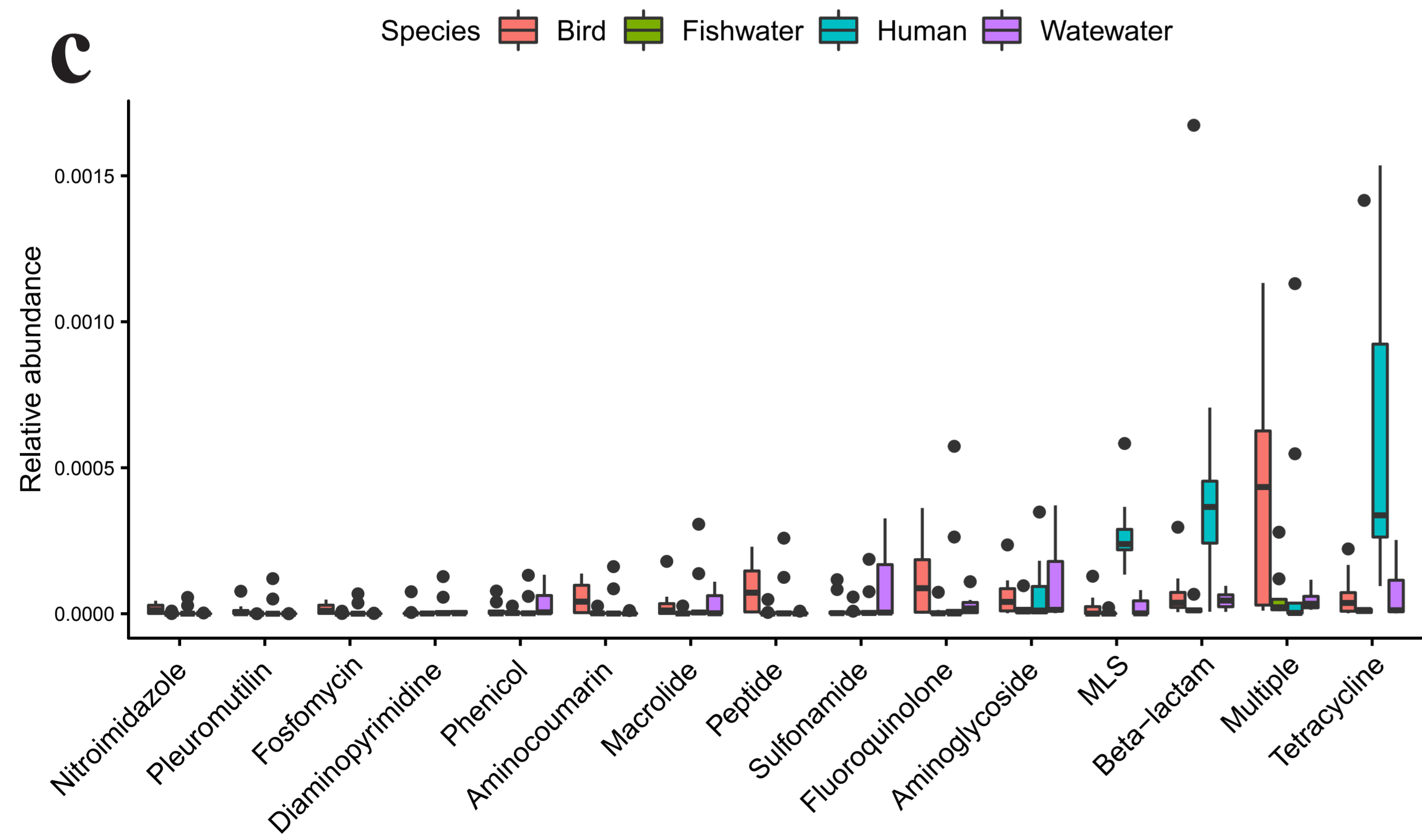**d**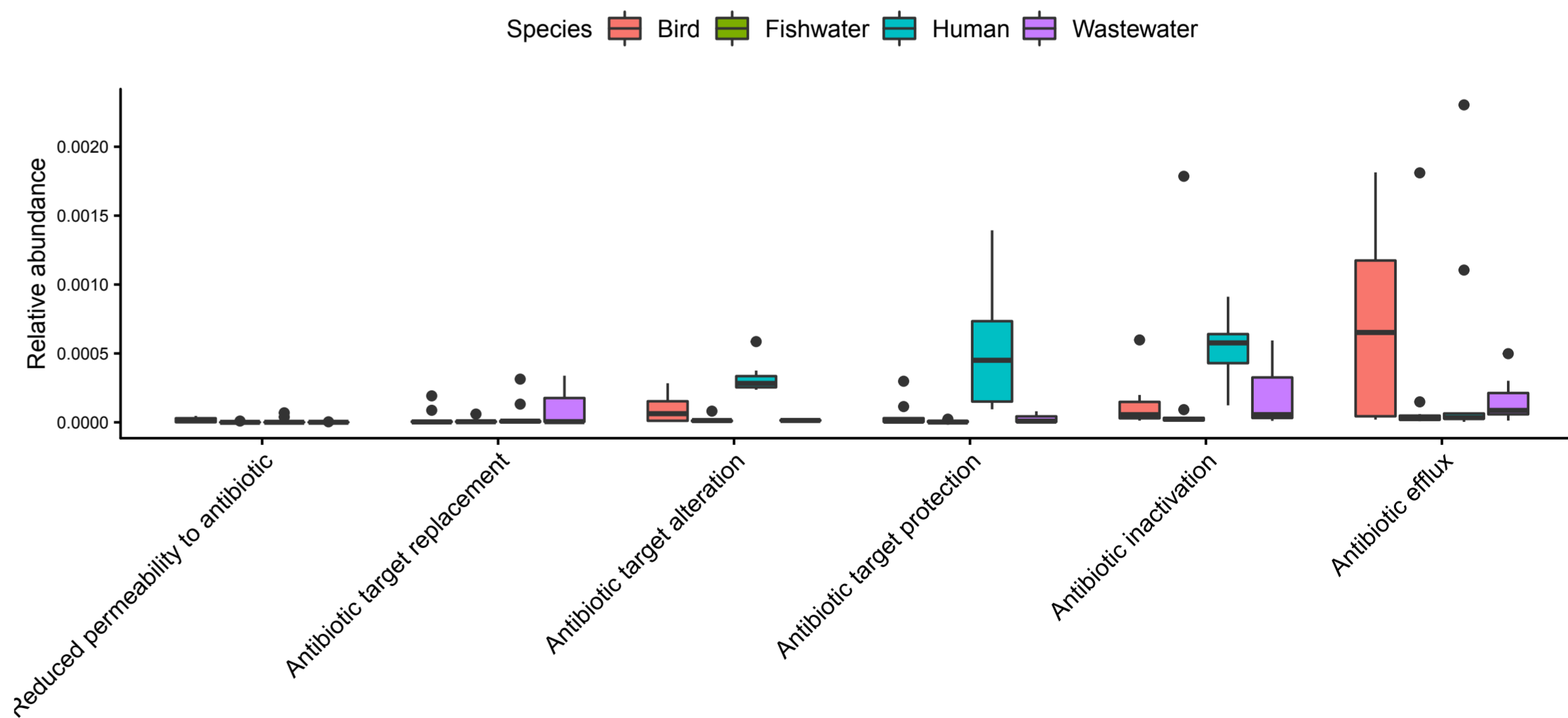

Supplement: Supplementary file 9 — Additional file 8: Figure S8. bird fecal and environmental microbiota and resistomes in Poyang Lake. (a) Relative abundances of microbial phyla, (b) Relative abundances of microbial family, (c) Absolute abundances of antibiotic resistance categories, (d) Absolute abundances of antibiotic resistance mechnism. Boxes denote the interquartile (IQR) between the first and third quartiles (25th and 75th percentiles, respectively) and the line inside denotes the median. Whiskers denote the lowest and highest values within 1.5 times and the IQR from the first and third quartiles, respectively. [file 40168_2019_781_MOESM8_ESM.pdf]
